# Experimental evidence on the impact of climate-induced hydrological and thermal variations on glacier-fed stream biofilms

**DOI:** 10.1093/femsec/fiae163

**Published:** 2024-12-14

**Authors:** David Touchette, Martina Gonzalez Mateu, Grégoire Michoud, Nicola Deluigi, Ramona Marasco, Daniele Daffonchio, Hannes Peter, Tom Battin

**Affiliations:** River Ecosystems Laboratory, Alpine and Polar Environmental Research Centre (ALPOLE), School of Architecture, Civil and Environmental Engineering (ENAC), École Polytechnique Fédérale de Lausanne, Sion, 1950, Switzerland; River Ecosystems Laboratory, Alpine and Polar Environmental Research Centre (ALPOLE), School of Architecture, Civil and Environmental Engineering (ENAC), École Polytechnique Fédérale de Lausanne, Sion, 1950, Switzerland; River Ecosystems Laboratory, Alpine and Polar Environmental Research Centre (ALPOLE), School of Architecture, Civil and Environmental Engineering (ENAC), École Polytechnique Fédérale de Lausanne, Sion, 1950, Switzerland; River Ecosystems Laboratory, Alpine and Polar Environmental Research Centre (ALPOLE), School of Architecture, Civil and Environmental Engineering (ENAC), École Polytechnique Fédérale de Lausanne, Sion, 1950, Switzerland; Biological and Environmental Sciences and Engineering Division (BESE), King Abdullah University of Science and Technology (KAUST), Thuwal, 23955-6900, Saudi Arabia; Biological and Environmental Sciences and Engineering Division (BESE), King Abdullah University of Science and Technology (KAUST), Thuwal, 23955-6900, Saudi Arabia; River Ecosystems Laboratory, Alpine and Polar Environmental Research Centre (ALPOLE), School of Architecture, Civil and Environmental Engineering (ENAC), École Polytechnique Fédérale de Lausanne, Sion, 1950, Switzerland; River Ecosystems Laboratory, Alpine and Polar Environmental Research Centre (ALPOLE), School of Architecture, Civil and Environmental Engineering (ENAC), École Polytechnique Fédérale de Lausanne, Sion, 1950, Switzerland

**Keywords:** biofilm, climate change, drought, glacier-fed stream, succession, warming

## Abstract

Climate change is predicted to alter the hydrological and thermal regimes of high-mountain streams, particularly glacier-fed streams. However, relatively little is known about how these environmental changes impact the microbial communities in glacier-fed streams. Here, we operated streamside flume mesocosms in the Swiss Alps, where benthic biofilms were grown under treatments simulating climate change. Treatments comprised four flow (natural, intermittent, stochastic, and constant) and two temperature (ambient streamwater and warming of +2°C) regimes. We monitored microbial biomass, diversity, community composition, and metabolic diversity in biofilms over 3 months. We found that community composition was largely influenced by successional dynamics independent of the treatments. While stochastic and constant flow regimes did not significantly affect community composition, droughts altered their composition in the intermittent regime, favouring drought-adapted bacteria and decreasing algal biomass. Concomitantly, warming decreased algal biomass and the abundance of some typical glacier-fed stream bacteria and eukaryotes, and stimulated heterotrophic metabolism overall. Our study provides experimental evidence towards potential and hitherto poorly considered impacts of climate change on benthic biofilms in glacier-fed streams.

## Teaser

Experimental changes to flow and temperature regimes in glacier-fed streams show that droughts alter biofilm microbial community composition, warming enhances carbon metabolism, and both reduce algal biomass.

## Introduction

Mountain glaciers hold a significant portion of the world's freshwater reserves (Immerzeel et al. 2020) and drive global-scale hydrological processes (Jansson et al. 2003). Glacier-fed streams (GFSs) deliver water, sediments, and nutrients to downstream aquatic ecosystems (Ren et al. 2019). Despite harsh environmental conditions, GFSs harbour diverse microbial life, primarily dominated by benthic biofilms (e.g. Wilhelm et al. 2013, Ezzat et al. 2022, Michoud et al. 2025). These biofilms play key roles in nutrient cycling, carbon fluxes, and primary production, and form the basis of food webs in stream ecosystems (Battin et al. 2016).

The GFS environment is dynamic, varying diurnally and seasonally (Smith et al. 2001, Hannah et al. 2007). Variations include changes in streamwater temperature (Williamson et al. 2019) and discharge (Kneib et al. 2020, Coviello et al. 2022), as well as suspended sediments that attenuate light and affect benthic algae (e.g. Boix Canadell et al. 2021). Additionally, seasonal shifts in water sources (e.g. glacier meltwaters *versus* groundwater) influence nutrient availability (Uehlinger et al. 2010). These environmental variations create unique conditions for freshwater biofilms to grow and promote microbial diversity (Besemer et al. 2007, Gautam et al. 2022).

Climate warming is expected to alter natural GFS environmental variations and to shift the timing of environmental windows beneficial for biofilm growth (Milner et al. 2017). In the European Alps, accelerated snowmelt and a 2.5°C per decade increase in streamwater temperature have been observed (Niedrist and Füreder 2021, Vorkauf et al. 2021). Moreover, benthic biofilms face stress from changing flow regimes, including prolonged and frequent droughts (Paillex et al. 2020) and high-flow events due to peak glacier melt or simply unpredictable rain events (Milner et al. 2017). Despite the impact of these environmental changes on GFS food web structure (Niedrist and Füreder 2018) and ecosystem respiration (Acuña et al. 2015, Leathers et al. 2024), for instance, our knowledge on how climate-induced warming and altered flow regimes affect GFS biofilm microbial composition and diversity remains limited.

While experimental manipulations are useful to assess impacts of environmental changes on microbial communities (e.g. Romero et al. 2019a, Li et al. 2023), such studies remain rare in the high mountains. The aim of our study was to experimentally assess how alterations of thermal and hydrological regimes influence GFS biofilm community composition and diversity. To this end, we experimented with streamside flume mesocosms in the Swiss Alps, growing biofilms under eight treatments that simulate climate change-driven environmental variations. Treatments combined four flow regimes with two temperature regimes. Flow regimes simulated (i) natural stream flow (natural), (ii) natural flow with frequent zero-flow droughts (intermittent), (iii) unpredictable variations (stochastic), and (iv) constant flow (constant). Temperature regimes simulated the ambient streamwater temperature (control) and warming (+2°C compared to control; warm). Over 103 days, we analysed microbial biomass and successional patterns, microbial diversity, and community structure using metabarcoding (16S and 18S rRNA genes). We identified microbial taxa that were either favoured or adversely impacted by the induced environmental variations and further assessed whether these changes affect biofilm microbial metabolic diversity using phenotypic microarray assays.

We hypothesized that variations in flow would affect biofilm community composition. Specifically, we anticipated that droughts cause mortality and affect both biomass and composition, that stochastic flow variations would restart microbial succession and maturation by eroding biofilms during high flows, and that a constant flow would decrease diversity by reducing environmental heterogeneity. Additionally, we hypothesized that elevated streamwater temperature reduces microbial diversity by decreasing the abundance of psychrophiles, generally prevailing in GFS (Bourquin et al. 2022). Our experiment allowed us to disentangle the effects of altered flow and temperature regimes on GFS biofilm structure and function.

## Methods

### Study site and experimental design

**Figure 1.**
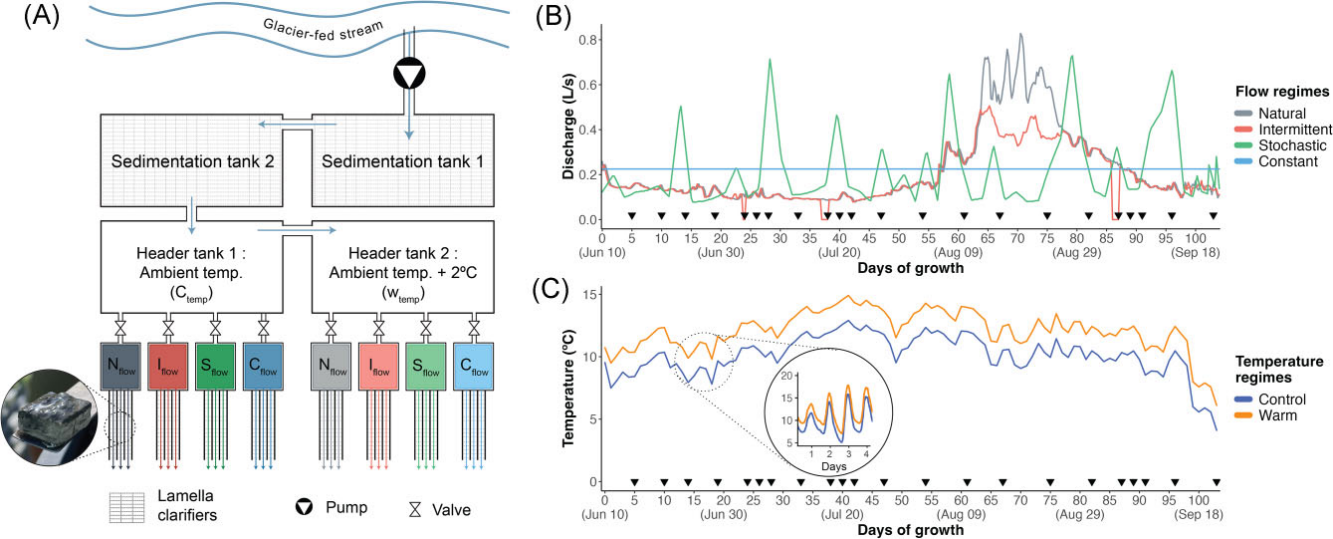
Experimental design and treatments. (A) Diagram of the experimental design. In brief, water from the GFS was pumped into sedimentation tanks before being transferred to the treatment header tanks. One of these tanks remained at the natural stream temperature (control treatments, C_temp_), while the other was consistently warmed by 2°C (warm treatments, *W*_temp_). For each treatment, a channel composed of three flumes allowed biofilm colonization and growth on clay coupons. The water discharge from the header tanks was controlled via valves, varying based on the different flow treatments: natural (N_flow_), intermittent (I_flow_), stochastic (S_flow_), and constant (C_flow_) regimes. (B) Graph of pre-determined discharge for each flow regime over time. (C) Graph of temperature for each temperature regime. The main graph shows the seasonal patterns of daily averaged temperature. The inset shows daily temperature variations. Arrows indicate sampling days (*n* = 22).

Flume mesocosms were constructed next to the *Dranse de Ferret*, draining the Val Ferret catchment (Swiss Alps; 45.906 N, 7.124 E; 1774 m above sea level) (Fig. 1). The catchment has been extensively studied and is one of the study sites of the Metabolism in Alpine Streams (METALP) monitoring project (https://metalp.epfl.ch). Mesocosms employed a factorial design to test the effects of ‘Succession’, ‘Flow’, ‘Temperature’, and ‘Treatment’ (for interaction of flow and temperature) on benthic biofilms, with four flow and two temperature regimes (Fig. 1). Flow treatments included a natural flow regime downscaling a typical hydrological year of *Dranse de Ferret* (from 2018 METALP data), a constant flow regime, a stochastic flow regime, and an intermittent flow regime. Stochastic flow regimes featured peak flows throughout the experiment to simulate high discharge due to unpredictable precipitation events. Intermittent flow featured three zero-flow periods simulating droughts (lasting 6 h on day 24, 24 h on day 38, and 24 h on day 87). Overall, flow regimes had an average discharge of 0.21 ± 0.13 L/s, an average velocity of 17.36 ± 2.75 cm/s, and an average channel water depth of 1.29 ± 0.56 cm over the experimental period (Figs 1 and S1). The experimental design also included an ambient temperature regime (i.e. control temperature) and a treatment mimicking a 2°C (1.98 ± 0.17°C) temperature increase compared to the control (i.e. warming). The experiment was conducted over 103 days from June to September 2022. Treatment and control flumes were operated in triplicates, resulting in 24 flumes overall.

Streamwater was continuously pumped from the *Dranse de Ferret* into two sedimentation tanks (2.55 m^3^ each) with lamella clarifiers (TUBEdek; ENEXIO Water Technologies GmbH), removing a fraction of the larger sediment particles to avoid heavy siltation on the biofilms; this kept streamwater turbidity in the tanks at a natural level (Fig. S2). Streamwater was then transferred into two header tanks (0.93 m^3^ each) fitted with temperature sensors (EMKO RTR-M06). One header tank was equipped with electrical screw immersion heaters, regulated to warm water temperature 2°C above background (i.e. control). Flumes were automatically fed from the header tanks, with the respective predetermined flow regimes regulated through motorized ball valves (TICOVAL; Ticom GmbH). Flumes (length: 250 cm; width: 3 cm; wall height: 5 cm) were constructed from polyvinyl chloride (PVC) walls mounted on a wooden surface, and had a 3% slope without a barrier at the flumes’ end. Each flume featured 85 unglazed, initially sterile clay coupons (2 × 2 × 0.33 cm) as substratum for biofilm growth, as done previously (e.g. Besemer et al. 2007). Flumes were sheltered by a transparent PVC roof to prevent flow alteration from heavy rain while ensuring light availability.

### Sample collection and physicochemical analysis

Coupons with benthic biofilms were used to estimate microbial community composition and diversity using metabarcoding (16S and 18S rRNA genes amplicon sequencing), as well as to determine bacterial cell abundance, chlorophyll-*a* as a proxy for algal biomass, and metabolic diversity. Coupons were collected every 4–5 days from June 15th to July 27th, subsequently every 7 days until August 31st, and again every 4–5 days until September 21st. Additional sampling was conducted three days after a drought event to better capture potential drought impacts on biofilms; this resulted in samplings one, three and five days after each drought. Sampling consisted of 22 sampling days in total. Coupons with biofilms collected for DNA and chlorophyll-*a* analyses were immediately flash-frozen on dry ice and stored at −80°C and −20°C, respectively, pending analyses. Coupons collected for the determination of bacterial abundance were transferred into a paraformaldehyde/glutaraldehyde solution (1% final concentration) supplemented with phosphate buffer solution (PBS, 0.01× final concentration), flash-frozen on dry ice, and stored at −80°C. In addition, the source community was sampled directly from the GFS by filtering 500 ml of streamwater (0.22 µm Sterivex filter), and filters were flash-frozen and stored at −80°C pending DNA extraction. At sampling times before and after droughts, an additional biofilm sample per flume was collected for metabolic potential assessment using EcoPlates (Biolog Inc.). These coupons were kept in 5 ml of filter-sterilized streamwater and maintained at 4°C until processing upon arrival in the laboratory (2 h on average). All sampling devices were flame-sterilized.

Streamwater and water from the header tanks were regularly collected for chemical analyses. Specifically, water temperature, pH, electrical conductivity, and dissolved oxygen concentration were measured using a SenTix^®^ 940-P pH electrode, a TetraCon^®^ 925 conductivity cell, and an FDO^®^ 925-P optical oxygen sensor connected to a MultiLine^®^Multi 3630 IDS reader (WTW Xylem Analytics). Turbidity was determined by averaging 10 measurements from a Turb^®^ 430 IR portable turbidity metre (WTW Xylem Analytics). Samples for major ion concentrations (ammonium, bromide, calcium, chloride, fluoride, lithium, magnesium, nitrate, nitrite, potassium, sodium, strontium, sulphate) were collected by filtering streamwater through a 0.2 µm polyethersulfone filter (Filtropur S; SARSTEDT) into 15 ml tubes and were kept at 4°C until analysis on a Metrohm 930 Compact IC Flex ion chromatography system. Samples for dissolved organic carbon (DOC) analysis were collected by filtering water through a pre-ashed GF/F filter (Whatman) into 40 ml acid-washed glass vials and were kept at 4°C for processing on a Sievers M9 TOC Analyzer (Veolia). These analyses indicated that streamwater chemistry did not vary markedly during the experiment (Fig. S1, Supplementary Table S1).

### DNA extraction and 16S/18S rRNA genes sequencing

Biofilms were scraped from coupons in 5 ml of ice-cold molecular-grade water, and the resulting slurry was centrifuged at 12 000 *g* (2 min). After removing 3.5 ml of supernatant, biofilm slurries were transferred into sterile tubes (1.5 ml) and centrifuged again to discard all remaining liquid. DNA was extracted from slurries using an optimized protocol (Busi et al. 2022), with an extra homogenization step involving bead beating (Precellys, Bertin Technologies) at 6000 r/m, with a 2 × 15 s and a 15 s break programme in 1.5 ml tubes containing 10–20% of 0.1 mm zirconium beads. Streamwater DNA was extracted from the Sterivex filters using a phenol-chloroform protocol described by Ezzat et al. (2022). DNA samples were quantified with the Qubit dsDNA HS kit (Invitrogen) and diluted to a final concentration between 0.9 and 3 ng/µl before amplification to limit PCR bias. Prokaryotic metabarcoding libraries targeting the 16S rRNA gene's V3–V4 hypervariable region were prepared using primers 341f (5′-CCTACGGGNGGCWGCAG-3′) and 785r (5′-GACTACHVGGGTATCTAATCC-3′) (Klindworth et al. 2013), and the eukaryotic libraries targeting the 18S rRNA gene's V4 loop were prepared using primers TAReuk454F (5′-TCGTCGGCAGCGTCAGATGTGTATAAGAGACAG-3′) and TAReukREV3 (5′-GTCTCGTGGGCTCGGAGATGTGTATAAGAGACAG-3′) (Stoeck et al. 2010). Amplification libraries were prepared according to the MiSeq manufacturer's protocol (Illumina), where amplicons were performed according to Ezzat et al. (2022) and verified on a 1.5% agarose gel. Libraries were normalized and sequenced on Illumina MiSeq using a 280-bp paired-end protocol at the Biological Core Lab, King Abdullah University of Science and Technology (KAUST, Saudi Arabia).

### Bioinformatics

In total, 1098 amplicon libraries from benthic biofilms and streamwater were generated (one sample was removed due to DNA extraction failure). Amplicon sequences were analysed using QIIME 2021.11 (Bolyen et al. 2019) with the default parameters. Briefly, raw sequences were demultiplexed and quality-based filtered using the q2-demux plugin, and q2-dada2 was used for denoizing with DADA2 (Callahan et al. 2016). Amplicon sequence variants (ASVs) taxonomy was assigned using q2-feature-classifier against the latest SILVA database v.138.1 (Quast et al. 2012), trained on the V3–V4 region for the 16S rRNA gene, and trained on the V4 loop for the 18S rRNA gene. Rarefaction curves, performed with the R *ranacapa* v.0.1.0 package (Kandlikar and Cowen 2018), ensured that ASV diversity reached saturation (Fig. S2). Non-bacterial ASVs (archaea, chloroplasts, mitochondria, eukaryotes), taxa with unassigned kingdom or without taxonomic assignment beyond kingdom were removed from the bacterial amplicon dataset for downstream analysis (Supplementary Table S2). Taxa from the Arthropoda phylum, and taxa with unassigned kingdom or without taxonomic assignment beyond kingdom were removed from the eukaryote amplicon dataset for downstream analysis (Supplementary Table S2). Then, singletons and ASVs only present in one sample were removed from the biofilm ASVs’ dataset (Supplementary Table S2). The eukaryotic dataset was divided into phototrophic (i.e. Bacillariophyta, Charophyta, Chlorokybophyceae, Chlorophyta, Cryptophyceae, Dinoflagellata, Florideophycidae, Haptophyta, Klebsormidiophyceae, Ochrophyta, Pavlovophyceae, Porphyridiophyceae, Prymnesiophyceae, Rhodellophyceae phyla) and non-phototrophic eukaryotes. ASVs from water and biofilm samples were compared using the *MicEco* v.0.9.19 R package (Russel 2021).

### Biomass quantification

Bacterial cell abundance was determined using flow cytometry according to Kohler et al. (2020). Biofilms were removed from coupons and slurries diluted with a fixative solution supplemented with tetrasodium pyrophosphate (0.025 mM final concentration), shaken (Standard Analog Shaker, VWR, 15 min, speed 5.5), sonicated (Sonifier 450, Branson, 1 min, 60% duty cycle, output 5), and transferred to new tubes. Coupons underwent a second round of cell removal, and both suspensions were combined, from which an aliquot was stained with SybrGreen^®^ (1× final concentration, incubation for 15 min at 37°C) and analysed on a volumetric flow cytometer (NovoCyte, ACEA Biosciences) equipped with a 488 nm laser. Three technical replicates were analysed per sample. Cell counts were corrected for the addition of fixatives and dyes and normalized to the coupon's surface area.

Phototrophic eukaryote biomass was estimated by quantifying chlorophyll-*a* content according to Kohler et al. (2020). In brief, 5 ml of 90% ethanol were added to tubes containing biofilm, and the tubes were placed into a water bath (78°C, 10 min) and incubated for 24 h (4°C in the dark). Tubes were then centrifuged, and the supernatant was measured on a microplate fluorometer reader (Synergy H1, Biotek) at 436/680 nm (ex./em.). Technical triplicates were analysed per coupon, and a spinach chlorophyll-*a* standard (Sigma Aldrich) was used to quantify biofilm chlorophyll-*a* content on an aerial basis. Together, bacterial cell abundance and phototrophic eukaryotic biomass provide a good proxy for biofilm biomass.

### Metabolic potential assessment

Potential biofilm metabolic diversity was assessed using 96-well Biolog EcoPlates containing triplicate panels of 31 unique carbon sources and a carbon-free control. Carbon substrate utilization was detected through the colorimetric change of the included tetrazolium violet redox indicator. Biofilms were removed from coupons, sonicated in a water bath (2 min), and centrifuged (2 min, 46 *g*) at 4 °C. The supernatant was transferred into a new tube, its optical density measured (OD_600_) on a microplate reader (Synergy H1, Biotek) and adjusted to 0.1 using filter-sterilized streamwater. For each flume, the diluted biofilm cell suspension was mixed, and 100 µl were inoculated into each well of one 31-carbon-source panel of the EcoPlate. Samples from the control temperature were incubated at 8°C, corresponding to the streamwater average temperature of the previous summer, while plates with samples from the warming treatment were incubated at 10°C to maintain the +2°C difference. OD_590_ was monitored three times weekly over 3 weeks. Measurements were normalized by subtracting the respective carbon-free control with resulting negative values set to zero, and absorbance values >0.25 considered positive (Yang et al. 2019). The number of different carbon substrates used (richness), Shannon carbon utilization diversity index, and carbon utilization evenness were calculated as per Yang et al. (2019) once average well colour development (AWCD) of each sample stabilized in time and reached a plateau (Yang et al. 2019, Touchette et al. 2022), which generally occurred after a 14-day incubation (Fig. S3).

### Data analysis and statistics

Analysis of alpha diversity and community composition estimations were performed with the R package *phyloseq* v.3.16 (McMurdie and Holmes 2013). Bacterial abundance, chlorophyll-*a*, and Shannon alpha diversity (bacterial and eukaryotic) data were tested for normality using the Shapiro–Wilk test from the R package *stats* v.4.3.0 (R Core Team 2020). To assess if ‘Succession’, ‘Flow’, ‘Temperature’, and ‘Treatment’ had a significant effect on biomass and diversity, the Kruskal–Wallis Rank Sum Test from the *stats* package was used. As ‘Flow’ was a significant predictor of chlorophyll-*a*, a pairwise comparison across flows was used to identify which specific flow regime was significantly different from the natural flow, using the Pairwise Wilcoxon Rank Sum Test from the R package *stats*.

Bacterial and eukaryotic community composition was explored with Bray–Curtis dissimilarity on Hellinger-transformed data, using the ‘vegdist’ and ‘transform’ functions of *vegan* v.2.6.4 (Oksanen et al. 2007) and *microbiome* v.1.24.0 (Lahti et al. 2017) R packages, respectively, and computed on a nonmetric multidimensional scaling (NMDS) using the ‘metaMDS’ function in *vegan*. The influence of the ‘Succession’ variable on community composition was modelled using the ‘ordisurf’ function from *vegan*, allowing for the estimation of smooth surfaces on the NMDS ordination. This creates contour lines by interpolating data points across the time intervals, representing predicted community compositions over succession time. The influence of measured environmental parameters was investigated by fitting the NMDS scores with the ‘envfit’ function, and the homogeneity of group dispersions was assessed with the ‘betadisper’ function, both in *vegan*. Collinearity between the environmental parameters and ‘Succession’ was assessed by variance inflation factor, using the ‘vif’ function of the *car* R package (Fox and Weisberg 2018). We assessed differences in microbial community composition based on ‘Succession’, ‘Flow’, ‘Temperature’, and ‘Treatment’ using the ‘adonis2’ function of *vegan* with 999 permutations and assessed the treatment effect using ‘pairwise.adonis’ from the *pairwiseAdonis* v.0.4.1 package (Martinez Arbizu 2020) with Benjamini–Hochberg adjusted *P-*values. The R package *ancombc* v.2.4.0 (Lin and Peddada 2024) was used on untransformed data to assess taxa differential abundance for each hydrological and thermal variation. The individual effect of each flow treatment was compared to the natural flow regime (e.g. natural flow warm temperature *versus* intermittent flow warm temperature, natural flow control temperature *versus* intermittent flow control temperature), and the effect of temperature was analysed in each individual flow treatment (e.g. natural flow control temperature *versus* natural flow warm temperature). This was performed with the ‘ancombc2’ function using ‘Succession’ and ‘FlumeID’ as random effects, the ‘bobyqa’ optimizer, and the Benjamini–Hochberg *P*-value adjustment method. Differential abundance was estimated at the genus level for bacteria and at the class level for eukaryotes (after agglomeration at the respective taxa levels). To be considered significantly differentially abundant, a taxon had to pass the *ancombc* sensitivity analysis test with an adjusted *P-value* < .05.

For each individual time-series (i.e. communities sampled over time from the same flume), the contribution of ecological processes to community assembly of bacterial communities was assessed using the binning-based phylogenetic framework iCAMP v.1.5.12, with a bin size of 48 (Ning et al. 2020). This uses the beta mean nearest taxon distance (ßNMTD) calculated from phylogenetic trees and taxonomic turnover including all bacterial ASVs (16S rRNA gene) present in a single flume, to partition the relative importance of homogeneous and heterogeneous selections, homogenizing dispersal, dispersal limitation as well as ecological drift. The relative contribution of these assembly processes individually was determined across all samples of each flume, and differences in assembly processes between treatments was assessed with the Tukey's Honest Significant Differences test from the *stats* package, after confirming normality of the ANOVA residuals using the Shapiro–Wilk test. Differences in functional metabolic diversity (substrate utilization richness, Shannon carbon utilization diversity index, and carbon utilization evenness) based on ‘Flow’ and ‘Temperature’ were assessed using the Kruskal–Wallis Rank Sum Test after testing for normality using the Shapiro-Wilk test. Data visualization was performed using the R packages *ggplot2* v.3.4.4 (Wickham 2016) and *microViz* v.0.10.10 (Barnett et al. 2021). Amplicon reads are available under the NCBI Bioproject PRJNA1086791. The code and data used in this study are available on the GitHub repository: https://github.com/RIVER-EPFL/Flume-mesocosms-biofilms.

## Results

### Dominant bacteria and eukaryotes in GFS mesocosm biofilms

**Figure 2.**
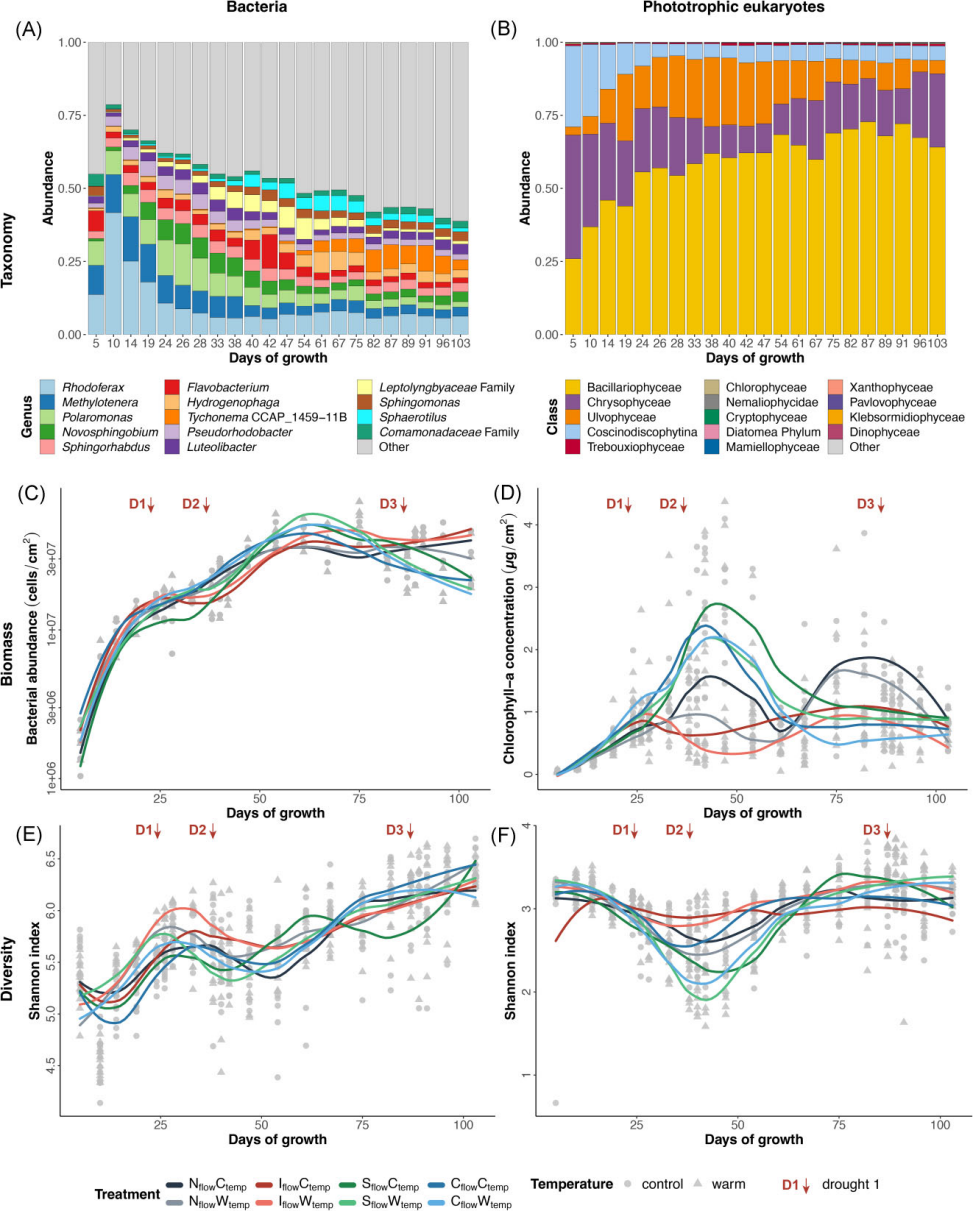
Microbial taxonomy, biomass, and diversity of GFS biofilm microbial members under climate change scenarios. Overall taxonomic composition of the (A) bacterial and (B) phototrophic eukaryotic communities, represented by the relative abundance of the 12 most abundant genera (bacteria) or classes (phototrophic eukaryotes). (C) Bacterial abundance reported as cells/cm^2^ and (D) chlorophyll-*a* concentration reported as µg/cm^2^, for each treatment and time point. Shannon alpha diversity index of (E) bacterial and (F) phototrophic eukaryotic communities for each treatment and time point. Lines represent the moving average, colour-coded per treatment. D1, D2, and D3 indicate the timing of the three droughts.

16S rRNA gene amplicon sequencing generated 17 286 unique ASVs from the benthic biofilms (27 841 595 reads after filtering). Overall, Pseudomonadota (previously Proteobacteria, 64.8 ± 11.1%), Bacteroidota (12.2 ± 3.7%), and Cyanobacteriota (previously Cyanobacteria, 8.0 ± 6.3%) phyla dominated biofilm communities (Fig. S5). *Rhodoferax* (10.2 ± 8.6%), *Methylotenera* (6.2 ± 3.9%), and *Polaromonas* (6.0 ± 3.8%) were the most abundant genera (Figs 2A and S5). Moreover, streamwater samples generated 16 326 bacterial ASVs (1 055 484 reads after filtering), of which 4856 were found in both biofilm and streamwater samples, representing a 14.4% overlap in prevalence of ASVs but 79.5% of the total community in terms of relative abundance (Figs S6 and S7).

18S rRNA gene amplicon sequencing generated 6400 ASVs from experimental biofilms (40 383 664 reads after filtering), of which 67.2% were phototrophic and 32.8% were non-phototrophic eukaryotes. Overall, phototrophic eukaryotes were dominated by Bacillariophyta (67.5 ± 10.3%), followed by Ochrophyta (18.3 ± 11.0%), and Chlorophyta (14.0 ± 9.0%) phyla (Fig. S8). At the class level, the dominant phototrophic eukaryotic taxa were Bacillariophyceae (59.5 ± 13.9%), Chrysophyceae (18.3 ± 11.0%), and Ulvophyceae (13.3 ± 9.2%) (Figs 2B and S8). Resolution at the genus level was poor as many ASVs were not identified down to the genus level, with *Hydrurus* (17.9 ± 11.1%) alongside *Diatoma* (14.4 ± 11.6%), as the main genera of the overall phototrophic eukaryotes (Fig. S8). Experimental biofilms were also populated by non-phototrophic eukaryotes, where the Cercozoa (26.5 ± 8.2%), Amoebozoa (19.1 ± 9.8%), and Ciliophora (15.8 ± 9.5%) phyla dominated the community (Fig. S9). Water samples generated 5957 eukaryotic ASVs (1 774 033 reads after filtering), and 2306 eukaryotic ASVs were found in both biofilm and water samples, representing an 18.7% overlap in prevalence of ASVs but 93.3% of the total community in terms of relative abundance (Figs S7 an S10).

### Flow variation and warming impacts on microbial biomass and diversity patterns

Temperature and flow variations differently affected biofilm biomass and diversity. Chlorophyll-*a* decreased under flow intermittency (*P* < .001) and warming (*P* = .009), with an interaction effect of drought and warming (*P* < .001) (Fig. 2D), and with a noticeable decrease following the first two droughts (D1, day 24 and D2, day 38). In contrast, flow, temperature, and their interaction did not significantly influence bacterial cell abundance (Fig. 2C). Bacterial and eukaryotic (both phototrophic and non-phototrophic) Shannon alpha diversity indices were also not impacted by warming and flow variations, nor by their interaction (Figs 2E–F and S11A). Succession was a significant predictor for all microbial biomass and diversity (*P* < .001). Overall, bacterial and eukaryotic (both phototrophic and non-phototrophic) communities tend to follow distinct succession stages (Nievas et al. 2022): a ‘colonization’ stage where biomass and diversity increased, ‘competition’ stages characterized by a decline in diversity, and ‘maturation’ stages with a shift towards more diverse communities (Figs 2E–F and S11A). This pattern was similar across all treatments, except for intermittent treatments where phototrophic eukaryotic communities did not show a clear ‘competition’ stage (Fig. 2F).

### Compositional changes induced by environmental variations

**Figure 3.**
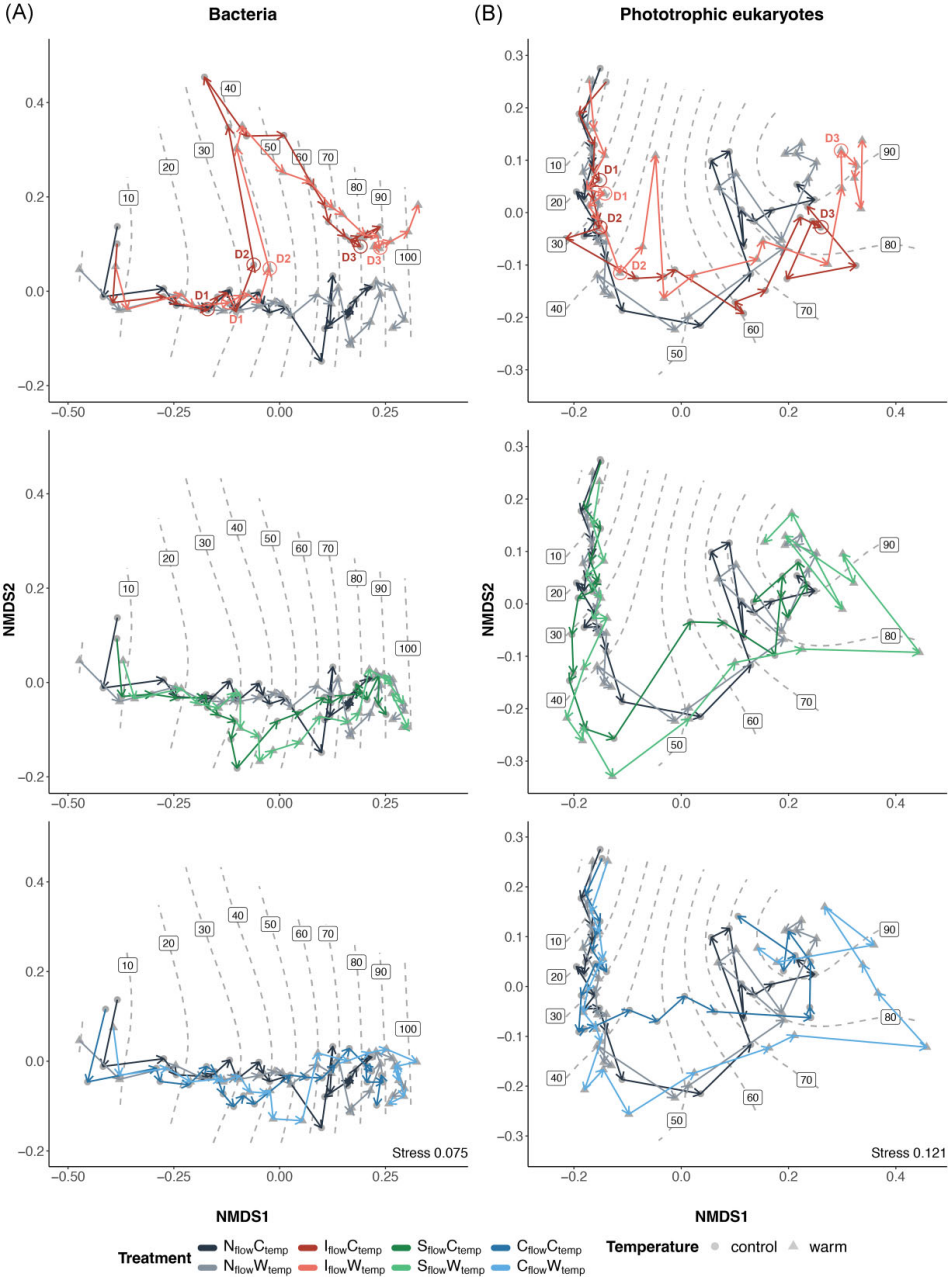
Microbial community composition of GFS biofilm microbial members under climate change scenarios. Changes in (A) bacterial and (B) phototrophic eukaryotic community composition are illustrated by NMDS ordination based on Bray–Curtis dissimilarity. Arrows indicate the ecological trajectories of biofilm samples over time. Top panels show differences between natural and intermittent flow regimes, where first time points after drought are indicated. Middle and bottom panels show differences from the natural flow regimes to the stochastic and constant flow regimes, respectively. Dashed contour lines represent time-based predictions (days) from the ‘ordisurf’ analysis, illustrating predicted community composition over succession time. Replicate samples were merged for visualization.

Exploring beta-diversity, we found that bacterial communities in all treatments differed primarily based on succession (*R*^2^ = 0.241, *P* < .001), followed by flow (*R*^2^ = 0.045, *P* < .001), and temperature (*R*^2^ = 0.012, *P* < .001) regimes, with a small treatment interaction effect (*R*^2^ = 0.006, *P* < .001). This was also shown by the first axis of the NMDS, which is strongly associated with succession, and the second axis of the NMDS being influenced by time since drought (Figs 3A and S12). A substantial increase in dissimilarity was observed on the NMDS after the second drought (D2, day 38), following which bacterial communities stayed dissimilar to other treatments over time (Fig. 3A, top panel). The eukaryotic communities mirrored this trend. Phototrophic eukaryotes differed across samples through succession (*R*^2^ = 0.255, *P* < .001), followed by flow (*R*^2^ = 0.024, *P* < .001) and temperature (*R*^2^ = 0.021, *P* < 0.001), with a small treatment interaction effect (*R*^2^ = 0.006, *P* < .001). Non-phototrophic eukaryotes differed across samples through succession (*R*^2^ = 0.230, *P* < .001), followed by flow (*R*^2^ = 0.021, *P* < .001), and minimally by temperature (*R*^2^ = 0.010, *P* < .001), and treatment interaction (*R*^2^ = 0.005, *P* < .001). While the NMDS pattern for the phototrophic eukaryotes was not as clear as for bacteria, a substantial increase in dissimilarity was also observed after the second drought (D2, day 38) for both eukaryotic communities (Figs 3B and S11B), especially for the non-phototrophic community of the intermittent warm treatment (Fig. S11B). Moreover, several environmental parameters could significantly explain variation in community composition, among which most were collinear with biofilm succession, such as turbidity, dissolved oxygen, and all ions except lithium (Figs S2 and S12–S14), increasing with time. Lastly, all biofilm microbial communities significantly differed (*P* < .001) from the streamwater communities (Fig. S15).

Pairwise comparisons revealed that the intermittent flow regime significantly changed bacterial (control temperature and warm temperature, *P* < .005), and both phototrophic (control temperature *P* < .01, warming temperature *P* < .05) and non-phototrophic eukaryotic (control temperature and warm temperature, *P* < .01) community composition (Supplementary Table S3). While constant flow did not modify microbial communities, only the stochastic flow regime in the warming treatment affected the composition of phototrophic communities (*P* < .05), compared to the warm natural flow treatment (Supplementary Table S3). Notably, bacterial and phototrophic eukaryotic communities in the warming treatments significantly differed from the control temperature treatments under all flow regimes (*P* < .05), but not the non-phototrophic eukaryotes (Supplementary Table S3).

### Differentially abundant microbial taxa under flow variations and warming

**Figure 4.**
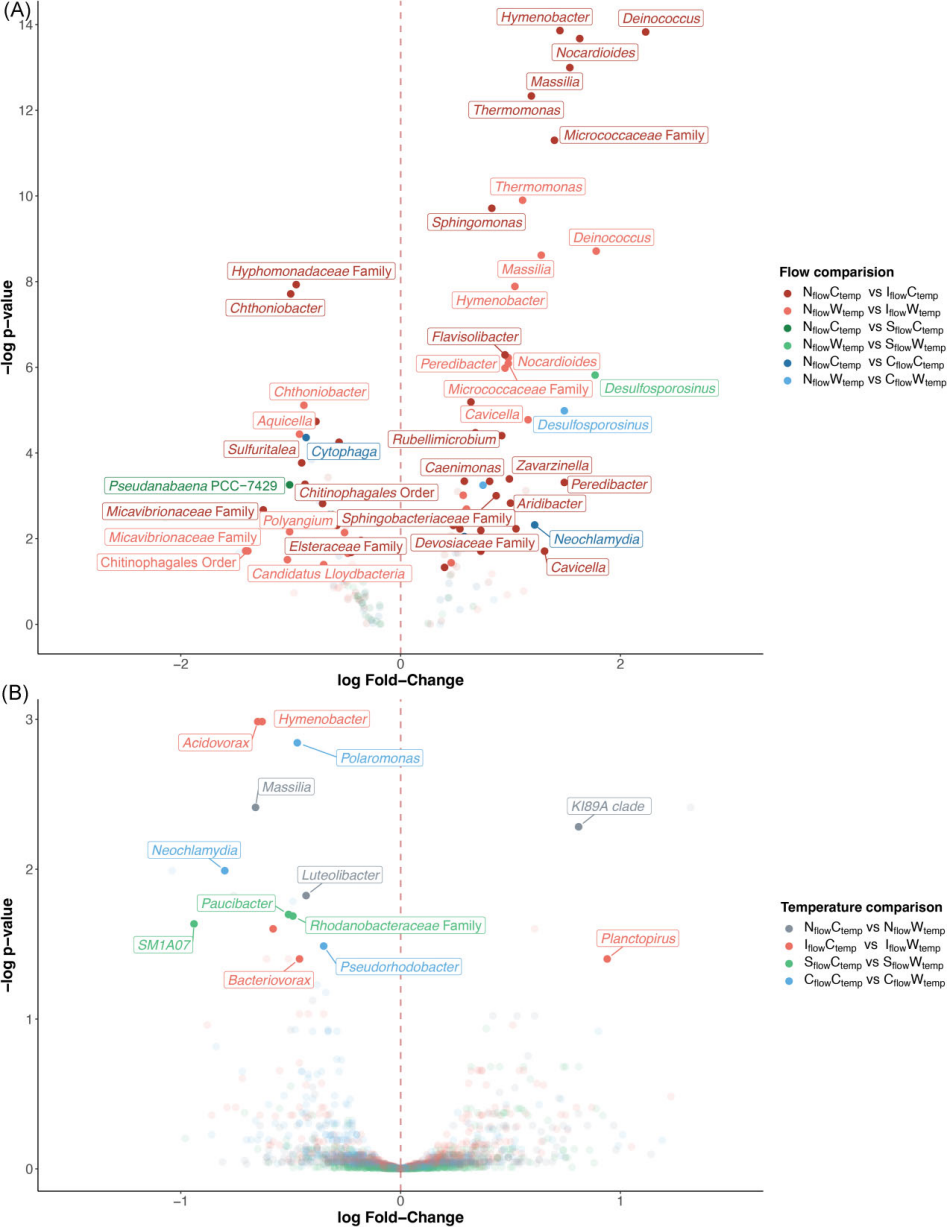
Differential abundance analyses showing the bacterial taxonomic differences between treatments and their respective control conditions. (A) Genera differentially abundant based on changing flow regimes. For visualization, only genera with a log fold-change higher than 0.8 and lower than −0.8 are labeled. Positive log fold-change values indicate an increase in taxa abundance in the I_flow_, S_flow_, and C_flow_ treatments relative to the N_flow_ treatments; negative log fold-change values indicate a decrease in abundance. (B) Genera differentially abundant based on changing temperature regimes. Positive and negative log fold-change values indicate an increase and a decrease (respectively) in taxa abundance in the warm treatments relative to the control temperature within the same flow regime.

We identified 62 bacterial genera across 12 phyla that were differentially abundant in the intermittent, stochastic, and constant flow treatments compared to the natural flow regime (Fig. 4A). Of these, 54 bacterial genera significantly changed in abundance (31 increased, 23 decreased) in intermittent treatments, but only 14 of them showed a consistent pattern in both temperature treatments: *Deinococcus, Nocardioides, Massilia, Peredibacter, Hymenobacter, Cavicella, Thermomonas, Sphingomonas, Parablastomonas*, and an unknown genus of the *Micrococcaceae* family increased in abundance, whereas *Luteolibacter, Chthoniobacter*, and unknown genera of the *Micavibrionaceae* family and Chitinophagales order decreased in abundance (Fig. 4A). The increase in abundance of the nine known genera in the intermittent flow regime is mostly triggered by the second drought (Fig. S16). In contrast, only eight and three bacterial genera changed in the constant flow and stochastic flow treatments, respectively; none of these changes occurred across both temperatures. When investigating temperature effects within each flow, differential abundance analysis identified 14 bacterial genera across 5 phyla as differentially abundant (2 increased, 12 decreased), but none showed a consistent response to warming across all flow regimes (Fig. 4B).

**Figure 5.**
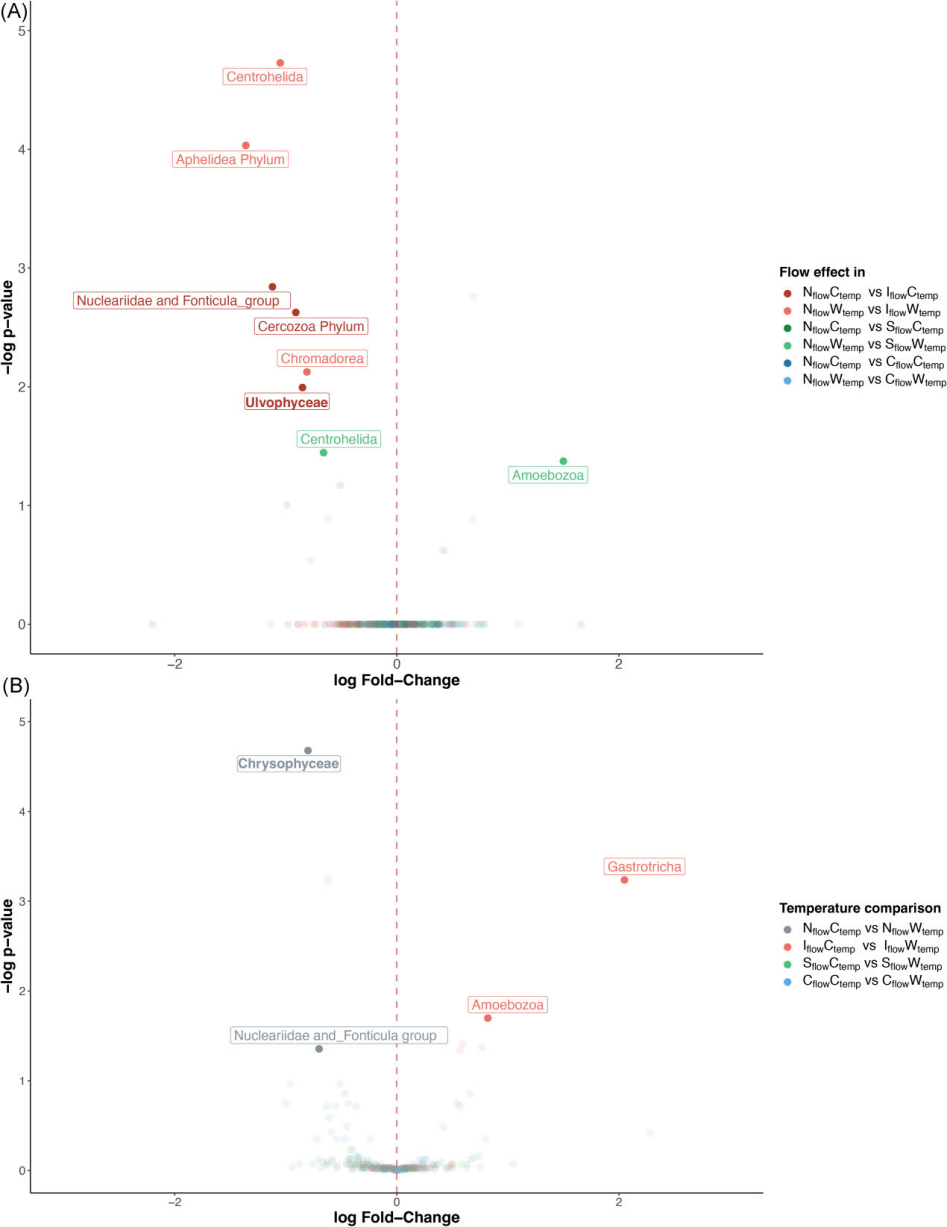
Differential abundance analyses showing the eukaryotic taxonomic differences between treatments and their respective control conditions. (A) Genera differentially abundant based on changing flow regimes. Positive log fold-change values indicate an increase in taxa abundance in the I_flow_, S_flow_, and C_flow_ treatments relative to the N_flow_ treatments; negative log fold-change values indicate a decrease in abundance. (B) Genera differentially abundant based on changing temperature regimes. Positive and negative log fold-change values indicate an increase and a decrease (respectively) in taxa abundance in the warm treatments relative to the control temperature within the same flow regime. Genera in bold are phototrophic eukaryotes, genera not in bold are non-phototrophic eukaryotes.

Comparing treatment effects on both eukaryotic communities, only seven classes, each from a different phylum, were found to be differentially abundant in the flow treatment compared to the natural regime, with intermittency being the main driver of those differences (Fig. 5A). Temperature-wise (Fig. 5B), Chrysophyceae and the unclassified ‘Nucleariidae and Fonticula group’ classes decreased in the natural flow under warming, while the classes Amoebozoa and Gastrotricha increased in the intermittent flow under warming. The limited number of eukaryotic taxa identified as differentially abundant might be attributable to the coarse taxonomic resolution of our analyses.

### Contribution of assembly processes to bacterial community assembly

**Figure 6.**
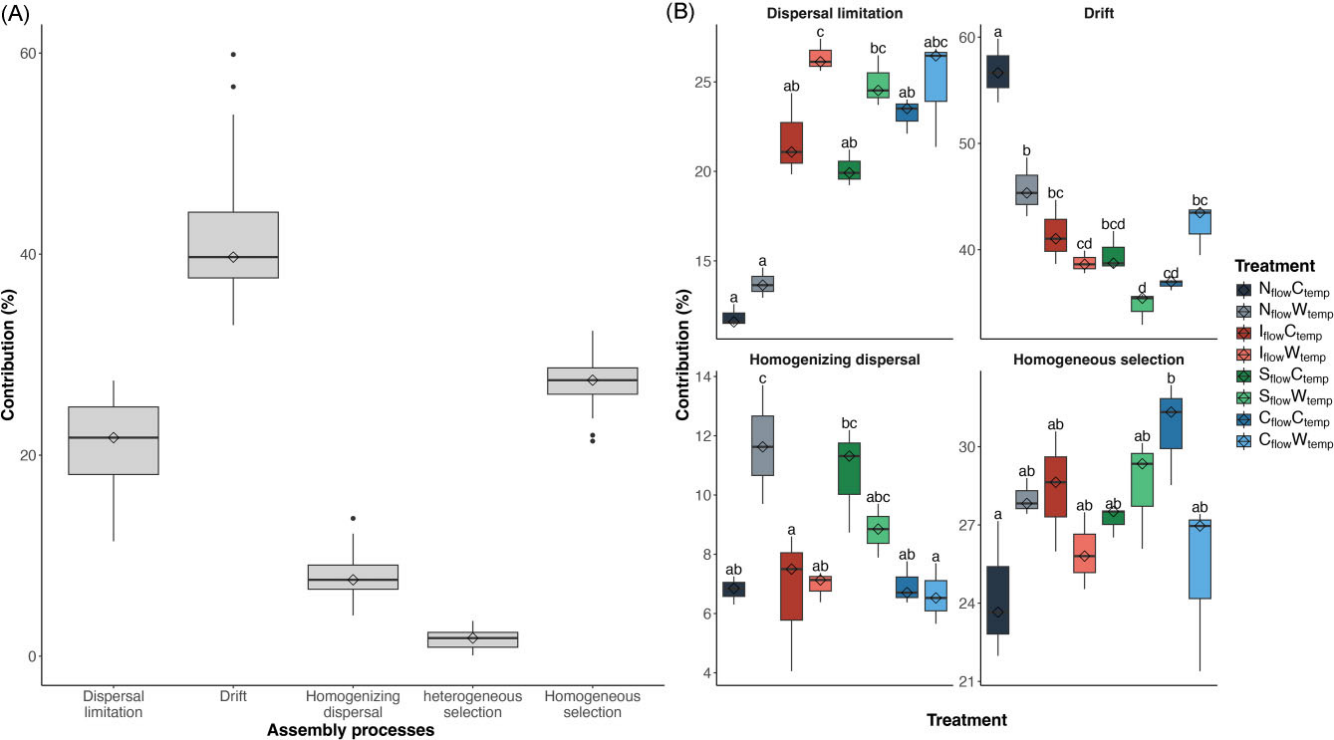
Assembly processes shaping GFS biofilms’ bacterial community assembly. (A) Overall contribution of dispersal limitation, drift, homogenizing dispersal, homogeneous selection, and heterogeneous selection on bacterial community assembly. (B) Contribution of dispersal limitation, drift, homogenizing dispersal, and homogeneous selection to bacterial community assembly across treatments. Letters indicate significant differences (*P* < .05) in each assembly process between treatments, as determined by Tukey's Honest Significant Differences test.

Overall, bacterial communities were mostly structured by ecological drift (42.0 ± 6.8% of the assembly processes), followed by homogeneous selection (27.3 ± 2.6%), dispersal limitation (20.9 ± 5.3%), homogenizing dispersal (8.2 ± 2.3%), and heterogeneous selection (1.7 ± 1.0%) (Fig. 6A). Notably, drift contributed more (*P* < .001) to community assembly in the natural flow with ambient temperature treatment (56.8 ± 3.0%) than in all other treatments (39.9 ± 3.9%) (Fig. 6B). Although not significant, dispersal limitation, or temporal persistence in the context of our time-resolved experimental design, was elevated in the warmed treatment for each flow regime (Fig. 6B). This was especially true when examining the changes in assembly process contribution of the intermittent differentially abundant known genera: *Deinococcus, Nocardioides, Massilia, Peredibacter, Hymenobacter, Cavicella, Thermomonas*, and *Sphingomonas* (Fig. S17).

### Treatment effects on metabolic diversity

**Figure 7.**
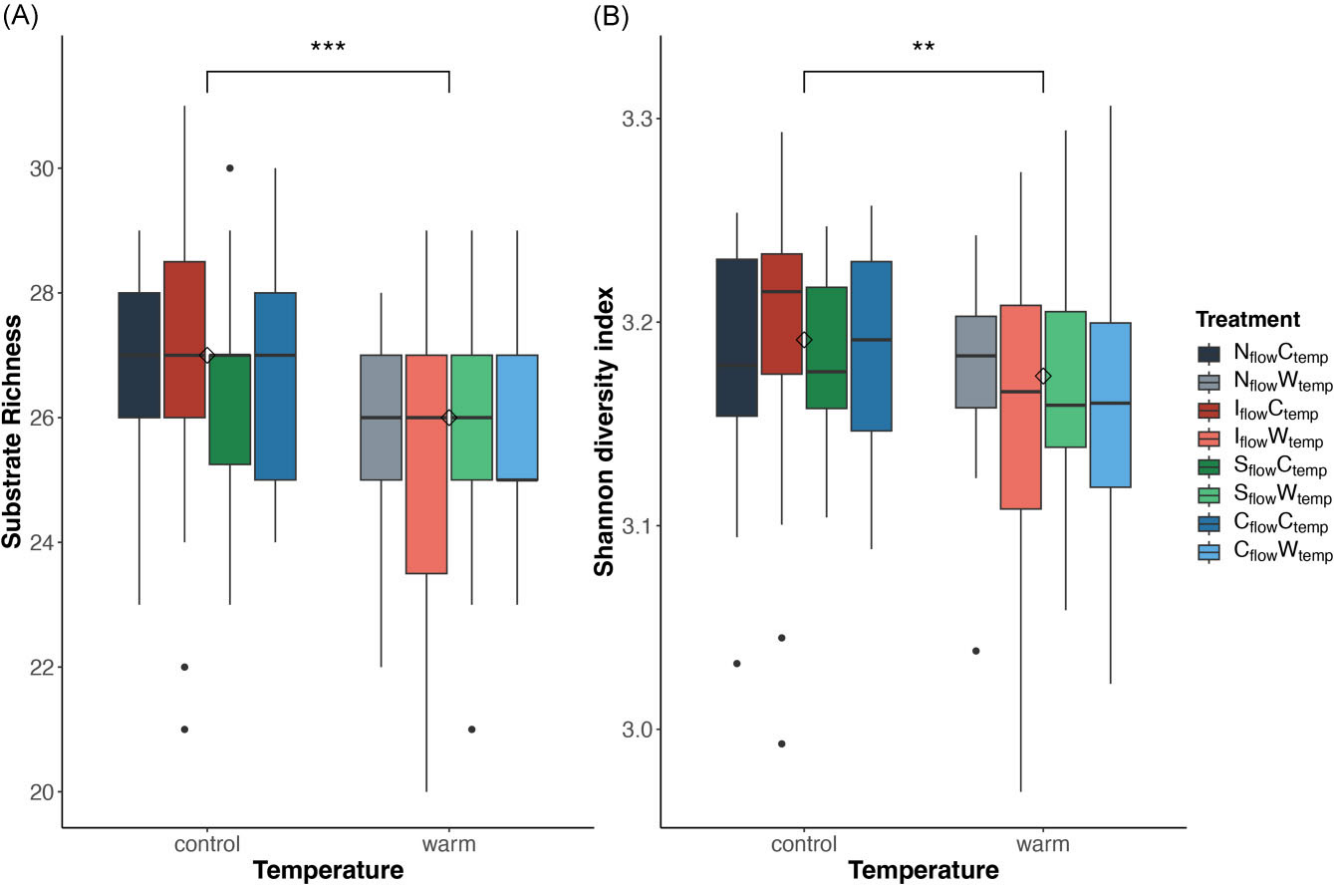
Microbial metabolisms in GFS biofilms. (A) Carbon utilization richness and (B) Shannon carbon utilization diversity index of GFS biofilm under different flow and temperature regimes. Significant differences between treatments are identified with stars (**: .01 > *P* > .005, ***: *P* < .005).

On average, biofilm microbial communities metabolized 26.4 ± 1.9 out of the 31 carbon substrates (Fig. 7), with an average Shannon carbon utilization diversity index of 3.16 ± 0.06 (Fig. 7), and carbon utilization evenness of 0.121 ± 0.007. No specific pattern of substrate utilization was observed, with the exception of temperature having a significant effect on metabolic diversity. Warming significantly increased the richness of substrates used (one carbon source on average, *P* < .001) and the Shannon carbon utilization diversity index (0.021 on average, *P* < .01), combined with a significant decrease in carbon utilization evenness (0.004 on average, *P* < .001). In brief, biofilm microorganisms metabolized a wide range of carbon substrates (high richness), but preferred few of them (low evenness).

## Discussion

Climate change is altering high-mountain stream ecosystems, with glacier shrinkage expected to impact the seasonality of environmental windows beneficial for biofilm growth (Milner et al. 2017). These alterations are thought to be caused, among other factors, by changes in streamwater discharge and warming related to decreasing glacier influence (e.g. Uehlinger et al. 2010, Boix Canadell et al. 2021).

Our analyses revealed that intermittent flow (i.e. zero-flow periods) impacted GFS biofilms the most, while stochastic and constant flow regimes did not show remarkable effects compared to natural stream conditions. Intermittency shifted bacterial and eukaryotic community compositions (both phototrophic and non-phototrophic) and decreased chlorophyll-*a* concentration. The absence of marked changes in the stochastic and constant flow regimes is tentatively attributed to GFSs being naturally subject to flow variations (Milner et al. 2017), such as at our experimental site (Boix Canadell et al. 2021), to which benthic biofilms have been naturally exposed, and likely adapted to. While the main channel of GFSs typically remains continuously open to flowing water (see multi-year data on https://metalp.epfl.ch), daily and nightly variations in discharge can cause areas near the banks (patches, habitats, and sediments) to dry out. Consequently, increasingly frequent intermittency within streams draining proglacial terrain (Paillex et al. 2020) may induce compositional shifts in aquatic microorganisms. We did not observe a compositional shift following the first short drought (6 h on day 24). However, a drastic alteration of the biofilm community was evident after the second, longer drought (24 h on day 38) (Fig. 3). This change is likely attributable to the biofilms’ capacity to retain moisture during shorter droughts (Gionchetta et al. 2019, Oprei et al. 2019), whereas prolonged no-flow periods lead to more extensive drying of the biofilms, thereby inducing greater community dissimilarity. These observations are consistent with other freshwater studies, which have shown that longer drought durations exacerbate resource limitations and osmotic stress, further affecting community composition (Romero et al. 2019a, Timoner et al. 2020, Li et al. 2023).

At the same time, intermittency was the only flow treatment resulting in consistently (across temperature regimes) differentially abundant bacteria (Fig. 4A). Specifically, four genera were less abundant under drought conditions, including *Chthoniobacter*, previously found to decrease in soils under drought stress (Dai et al. 2019). However, 10 genera were enriched in the intermittent flow treatment (Fig. 4A), strongly linked to the second drought period (24 h on day 38; Fig. S16). Among them, genera often associated with harsh and dry conditions were present. These included the multi-resistant *Deinococcus, Hymenobacter*, and *Sphingomonas* genera, capable of forming biofilms on polar solar panels (Tanner et al. 2018), the desiccation-resistant *Massilia* (Gruppuso et al. 2023), and the ionizing radiation-resistant genus *Nocardioides* (Yu et al. 2015), which has been shown to survive atmospheric transport of desert dust (Griffin 2007). Surprisingly, spore formation does not seem to be common in these genera enriched under drought conditions, a feature often associated with drought resistance (Sabater et al. 2016a,b). However, these enriched genera are known to produce extracellular polymeric substances (EPSs) (Baker et al. 2010, Reuben et al. 2012, Meneghine et al. 2017), which can retain water within biofilms, overcoming desiccation (Flemming and Wingender 2010). Furthermore, drought in stream ecosystems tends to shift sediment biofilms towards those found in drier environments like soils, favouring members of the Alphaproteobacteria and Actinomycetes (previously Actinobacteria) classes (Pohlon et al. 2018, Romero et al. 2019b, Foulquier et al. 2024). Accordingly, 5 out of the 10 genera favoured during drought belong to these bacterial classes, suggesting that as GFSs dry out, they may start to resemble surrounding dry sediments.

Therefore, significant bacterial composition changes in the intermittent flow following the second drought (Fig. 3A) were likely due to a filtration effect towards drought-tolerant taxa and EPS producers, allowing the biofilm to withstand subsequent droughts. Such adaptations to repetitive droughts were previously observed in streams (Timoner et al. 2020). This is perhaps why the third drought (24 h on day 87) did not substantially increase community dissimilarity in our GFS biofilms, as they remained different from other flow treatments (Fig. 3A). Moreover, all treatments appeared to reduce the contribution of drift to bacterial community assembly (Fig. 6B), suggesting that climate change could reduce the importance of stochastic processes in shaping microbial communities of GFS biofilms. Despite these marked compositional changes, intermittency did not impact carbon source utilization (Fig. 7), supporting the observed functional redundancy in GFS (Wilhelm et al. 2015, Busi et al. 2022).

Despite the significant change in community dissimilarity (Fig. 3B), we did not observe a clear pattern of flow treatment effect on taxonomic differential abundance in eukaryotic communities (Fig. 5). This finding suggests that GFS biofilm eukaryotes may be more resilient to drought, possibly due to the ability of many of them to enter dormancy, especially diatoms, when faced with unfavourable conditions (Simon et al. 2016, Sabater et al. 2016b), or due to the analysis being performed at a broad taxonomic level (order). The higher resilience of eukaryotes to drought compared to bacteria is corroborated by previous studies on stream biofilms demonstrating faster recovery of primary production compared to respiration after rewetting (Acuña et al. 2015, Zlatanović et al. 2018). Eukaryotic resilience to drought could result from structural features like scaffolding or drought-resistance-enhancing mucilage by diatoms, such as *Diatoma* and *Cymbella* (Sabater et al. 2016b, Novais et al. 2020), dominant diatoms in GFS biofilms (Fig. S8), or simply by their ability to migrate to wetter parts of the biofilm (McKew et al. 2011).

Though less pronounced than drought, warming also modified the community composition of bacteria and phototrophic eukaryotes in GFS biofilms across all flow regimes (Supplementary Table S3). This is consistent with previous work on non-GFS biofilms, where hydrological disturbances had stronger impacts on composition than warming (Romero et al. 2019a). Interestingly, such an impact of temperature was not observed for the non-phototrophic eukaryotes (Supplementary Table S3), suggesting they are more tolerant to temperature changes. Despite an overall significant shift in beta diversity with warming, only a few microbial taxa were found differentially abundant based on temperature, and none of them were consistent across the flow regimes (Figs 4B and 5B). Noteworthy, we found Chrysophyceae (Fig. 5B), mostly represented by the *Hydrurus* genus, less abundant in the natural flow regime warmed to +2°C. This suggests that the cryophilic photosynthetic algae *Hydrurus* (Klaveness 2019), iconic to high-mountain streams, may decrease in abundance as streamwater temperature rises (Jorgenson et al. 2024). The overall limited response of biofilm microbial composition to warming of +2°C aligns with the mesophilic character of many GFS bacteria, as recently suggested by genomic evidence (Michoud et al. 2025). This also makes sense, given the marked daily and seasonal temperature variations in GFSs (Fig. 1). Moreover, carbon utilization diversity was enhanced by warming (Fig. 7), supporting a temperature-dependent stimulation of biofilm carbon degradation, consumption, and respiration (Ylla et al. 2014, Freixa et al. 2017, Romero et al. 2019a).

We found that overall algal biomass significantly decreased with both intermittency and warming in GFS biofilms, and more when combined (Fig. 2D). This response to drought aligns with previous studies on non-GFS biofilms, which observed a decrease in chlorophyll-*a* concentration, gross primary production, and photosynthetic yield after desiccation (Colls et al. 2019, Nelson et al. 2023). Such a reduction in chlorophyll-*a* could originate from drought-induced osmotic stress, which can damage the photosynthetic machinery and cause cellular lysis (Sabater et al. 2016a). It is also possible that phototrophs decrease chlorophyll-*a* in favour of accompanying pigments (e.g. carotenoids) that protect them against oxidative stress. This strategy is an adaptative mechanism to limit energy loss to allow cell repair (Sabater et al. 2016b). Moreover, droughts (DeColibus et al. 2017) and warming (Villanueva et al. 2011, Freixa et al. 2017, Sudlow et al. 2023) can both individually favour grazers in biofilms. Accordingly, we observed an elevated abundance of eukaryotic biofilm grazers, Gastrotricha and Amoebozoa (Kulishkin et al. 2023, Manirakiza et al. 2024), in the intermittent flow treatment under warming (Fig. 5B). The differential increase in these eukaryotic grazers could also be responsible for the decreased phototrophic biomass (Fig. 2D). Biofilms exposed to drought and warming contained the lowest chlorophyll-*a* concentrations compared to other treatments, potentially due to the combined effect of cellular lysis, pigment reshuffling, and grazing.

Over the experiment, mesocosm biofilms followed typical stages of succession (i.e. colonization, competition, and maturation) (Jackson 2003, Veach et al. 2016, Brislawn et al. 2019) had microbial taxa normally found in GFSs (Brandani et al. 2022), and differed from the streamwater (Brandani et al. 2022, Ezzat et al. 2022), suggesting the experimental set-up was able to replicate *in-situ* conditions consistent with environmental GFS biofilms. Succession had a significant effect on microbial biomass, alpha diversity, and community composition (Fig. 2E, F, and S11), reflecting the strong influence of temporal patterns in microbial stream biofilms (Gautam et al. 2022). This is also suggested by the high contribution of drift to bacterial community assembly (Fig. 6A), a stochastic process that arises from temporal influences (Bier et al. 2022). Surprisingly, microbial diversity did not stabilise, even after 103 days of growth. This could be due to changing water sources and conditions in GFSs, which could promote microbial diversity (Wilhelm et al. 2013). As most of our measured environmental parameters could significantly explain changes in community composition and most of them were collinear with biofilm succession (Figs S2 and S12–S14), it is possible that streamwater origin proportions changed throughout the season, creating new environmental conditions within the mesocosms. As beta diversity is influenced by the physical constraints of streams, such as sediment transport and nutrient availability (Tolotti et al. 2024), these environmental changes potentially further favoured the development of different niches for new microorganisms to establish (Besemer et al. 2007) and, therefore, promoted dissimilarity between the communities through time (Figs 3 and S11). This is further supported by the overall high contribution of homogeneous selection to community assembly (Fig. 6A).

## Conclusion

Biofilm communities exhibited strong successional turnover despite the climatic pressures we simulated in this experiment. While stochasticity and flow homogenization did not affect the diversity and composition of GFS biofilm communities, extended zero-flow periods (intermittency) favoured drought-adapted bacteria while reducing algal biomass and the abundance of other microbial taxa. Warming of streamwater by 2°C also altered benthic biofilms by decreasing phototrophic biomass and the abundance of some natural GFS microorganisms, like the iconic *Hydrurus*, while selecting for biofilm grazers and stimulating the assimilation of carbon substrates. Overall, our experimental study has revealed flow variations and warming impacts on GFS biofilms, which may be important for better understanding GFSs under a changing climate.

## Supplementary Material

fiae163_Supplemental_File
