# Supplementary material for: Experimental evidence on the impact of climate-induced hydrological and thermal variations on glacier-fed stream biofilms
Source: FEMS Microbiol Ecol. 2024 Dec 14;101(1):fiae163. doi: 10.1093/femsec/fiae163 (PMC11705997; doi:10.1093/femsec/fiae163)
Supplement: fiae163_Supplemental_File [file fiae163_supplemental_file.docx]

**Supplementary Table 1. Averaged environmental parameters measured during sampling**. Values represent the average of 22 sampling days, and their standard deviations. In brief, water temperature, pH, electrical conductivity, and dissolved oxygen (O_2_) concentration were measured using a SenTix® 940-P pH electrode, a TetraCon® 925 conductivity cell, and an FDO® 925-P optical oxygen sensor connected to a MultiLine® Multi 3630 IDS reader (WTW Xylem Analytics). Turbidity was determined by averaging 10 measurements from a Turb® 430 IR portable turbidity metre (WTW Xylem Analytics). Ion chromatography was performed on a Metrohm 930 Compact IC Flex. Dissolved organic carbon (DOC) was processed on a Sievers M9 TOC Analyzer (Veolia). Bold text with a star (*) indicates that the measured environmental parameter was significantly different (*p* < 0.05) from that in the streamwater compared to the two other experimental conditions.

| **Environmental parameter** | ***Dranse de Ferret* streamwater** | **Control temperature header tank** | **Warm temperature header tank** |
| --- | --- | --- | --- |
| Ammonium  (ppm) | 0.014 ± 0.0050 | 0.014 ± 0.00065 | 0.013 ± 0.00055 |
| Bromide  (ppm) | 0.0025 ± 0.00085 | 0.0025 ± 0.00076 | 0.0027 ± 0.00095 |
| Conductivity  (µS/cm) | 365.95 ± 40.84 | 366.64 ± 40.02 | 367.32 ± 40.12 |
| Calcium  (ppm) | 61.10 ± 6.69 | 61.30 ± 6.56 | 61.45 ± 6.49 |
| Chloride  (ppm) | 0.37 ± 0.14 | 0.37 ± 0.14 | 0.37 ± 0.14 |
| DOC  (ppm) | 0.15 ± 0.041 | 0.14 ± 0.033 | 0.15 ± 0.040 |
| Fluoride  (ppm) | 0.012 ± 0.0015 | 0.012 ± 0.0014 | 0.012 ± 0.0013 |
| Lithium  (ppm) | 0.011 ± 0.0021 | 0.010 ± 0.0021 | 0.011 ± 0.0021 |
| Magnesium  (ppm) | 8.4 ± 1.1 | 8.4 ± 1.1 | 8.5 ± 1.1 |
| Nitrate  (ppm) | 0.39 ± 0.25 | 0.34 ± 0.024 | 0.34 ± 0.023 |
| Nitrite  (ppm) | 0.0037 ± 0.0090 | 0.0038 ± 0.0042 | **0.0045 ± 0.0030*** |
| O_2_  (mg/L) | 9.76 ± 0.54 | 9.78 ± 0.54 | 9.68 ± 0.49 |
| pH | **8.42 ± 0.06*** | 8.34 ± 0.13 | 8.33 ± 0.07 |
| Potassium  (ppm) | 0.36 ± 0.35 | 0.30 ± 0.077 | 0.33 ± 0.19 |
| Sodium  (ppm) | 1.35 ± 0.31 | 1.33 ± 0.25 | 1.35 ± 0.27 |
| Strontium  (ppm) | 0.70 ± 0.096 | 0.71 ± 0.089 | 0.71 ± 0.098 |
| Sulphate  (ppm) | 79.50 ± 13.67 | 79.78 ± 13.45 | 79.71 ± 13.52 |
| Temperature (ºC) | 8.08 ± 2.0 | 8.29 ± 1.84 | **10.5 ± 1.89*** |
| Turbidity (NTU) | 62.50 ± 80.33 | 47.01 ± 30.86 | 50.60 ± 33.92 |

**Supplementary Table 2. Filters used and number of ASV removed prior to biofilm microbial communities’ data analysis.**

| **Bacteria (16S rRNA gene)** | |
| --- | --- |
| **Filters** | **Filtered ASVs** |
| Unassigned kingdom / no assignment beyong kingdom | 1,272 |
| Chloroplast | 4,072 |
| Mitochondria | 232 |
| Archaea | 81 |
| Eukaryote | 8 |
| Singletons / only in one sample | 34,203 |
| **Eukaryotes** **(18S rRNA gene)** | |
| **Filters** | **Filtered ASVs** |
| Unassigned kingdom / no assignment beyong kingdom | 6,421 |
| Arthropoda | 592 |
| Bacteria | 4 |
| Singletons / only in one sample | 12,965 |

**Supplementary Table 3. Results of “pairwise.adonis”, assessing treatment effects on microbial composition, compared to their respective controls**. Bold text with a star (*) indicates a significant difference (*p* < 0.05) between the treatment and its control.

| **Bacteria (16S rRNA gene)** | | | |
| --- | --- | --- | --- |
| **Flow comparisons  (to natural flow regime)** | **F.Model** | **R^2^** | **Adjusted p value** |
| N_flow_C_temp_ vs I_flow_C_temp_ | 8.122 | 0.059 | **0.002*** |
| N_flow_W_temp_ vs I_flow_W_temp_ | 6.084 | 0.045 | **0.002*** |
| N_flow_C_temp_ vs S_flow_C_temp_ | 1.634 | 0.013 | 0.094 |
| N_flow_W_temp_ vs S_flow_W_temp_ | 1.765 | 0.013 | 0.068 |
| N_flow_C_temp_ vs C_flow_C_temp_ | 1.985 | 0.015 | 0.075 |
| N_flow_W_temp_ vs C_flow_W_temp_ | 1.408 | 0.011 | 0.132 |
| **Temperature comparisons  (in each flow)** | **F.Model** | **R^2^** | **Adjusted p value** |
| N_flow_C_temp_ vs N_flow_W_temp_ | 2.424 | 0.018 | **0.023*** |
| I_flow_C_temp_ vs I_flow_W_temp_ | 2.209 | 0.017 | **0.034*** |
| S_flow_C_temp_ vs S_flow_W_temp_ | 2.551 | 0.019 | **0.027*** |
| C_flow_C_temp_ vs C_flow_W_temp_ | 2.467 | 0.019 | **0.027*** |
| **Phototrophic eukaryotes (18S rRNA gene)** | | | |
| **Flow comparisons  (to natural flow regime)** | **F.Model** | **R^2^** | **Adjusted p value** |
| N_flow_C_temp_ vs I_flow_C_temp_ | 3.567 | 0.027 | **0.007*** |
| N_flow_W_temp_ vs I_flow_W_temp_ | 2.614 | 0.020 | **0.033*** |
| N_flow_C_temp_ vs S_flow_C_temp_ | 1.777 | 0.014 | 0.108 |
| N_flow_W_temp_ vs S_flow_W_temp_ | 3.328 | 0.0250 | **0.016*** |
| N_flow_C_temp_ vs C_flow_C_temp_ | 1.490 | 0.011 | 0.138 |
| N_flow_W_temp_ vs C_flow_W_temp_ | 1.193 | 0.009 | 0.273 |
| **Temperature comparisons  (in each flow)** | **F.Model** | **R^2^** | **Adjusted p value** |
| N_flow_C_temp_ vs N_flow_W_temp_ | 3.021 | 0.023 | **0.015*** |
| I_flow_C_temp_ vs I_flow_W_temp_ | 2.913 | 0.022 | **0.015*** |
| S_flow_C_temp_ vs S_flow_W_temp_ | 4.496 | 0.034 | **0.007*** |
| C_flow_C_temp_ vs C_flow_W_temp_ | 4.151 | 0.031 | **0.007*** |
| **Non-phototrophic eukaryotes (18S rRNA gene)** | | | |
| **Flow comparisons  (to natural flow regime)** | **F.Model** | **R^2^** | **Adjusted p value** |
| N_flow_C_temp_ vs I_flow_C_temp_ | 3.164 | 0.024 | **0.009*** |
| N_flow_W_temp_ vs I_flow_W_temp_ | 2.901 | 0.0218 | **0.005*** |
| N_flow_C_temp_ vs S_flow_C_temp_ | 1.037 | 0.008 | 0.365 |
| N_flow_W_temp_ vs S_flow_W_temp_ | 1.783 | 0.014 | 0.071 |
| N_flow_C_temp_ vs C_flow_C_temp_ | 1.405 | 0.011 | 0.162 |
| N_flow_W_temp_ vs C_flow_W_temp_ | 0.936 | 0.007 | 0.447 |
| **Temperature comparisons  (in each flow)** | **F.Model** | **R^2^** | **Adjusted p value** |
| N_flow_C_temp_ vs N_flow_W_temp_ | 1.644 | 0.012 | 0.095 |
| I_flow_C_temp_ vs I_flow_W_temp_ | 1.706 | 0.013 | 0.076 |
| S_flow_C_temp_ vs S_flow_W_temp_ | 2.331 | 0.018 | 0.058 |
| C_flow_C_temp_ vs C_flow_W_temp_ | 1.918 | 0.015 | 0.072 |

**
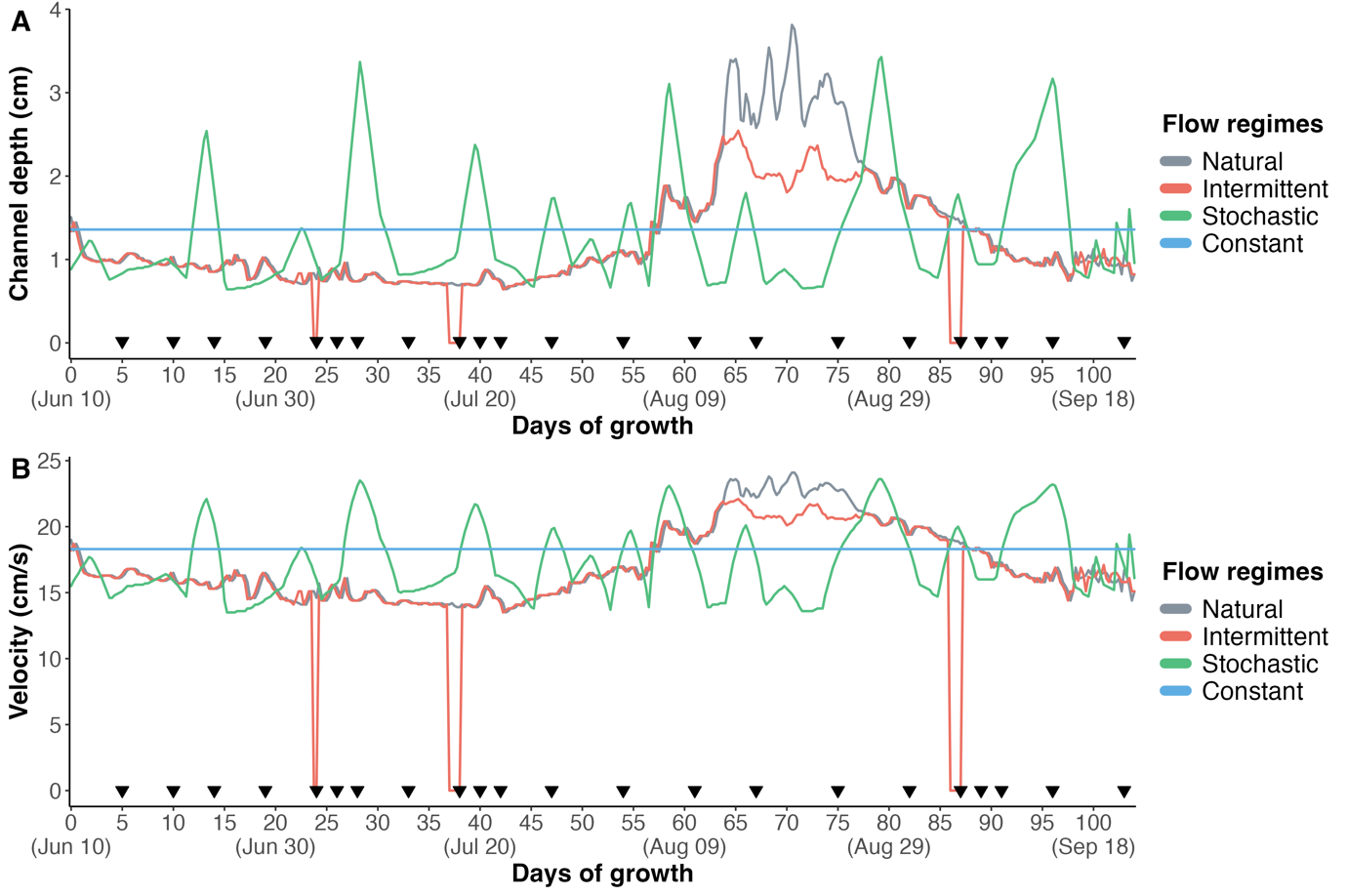
**

**Figure S1. Channel depth and velocity in experimental flow treatments.** Graphs of pre-determined **(A)** channel water depth and **(B)** velocity for each flow regime over time.


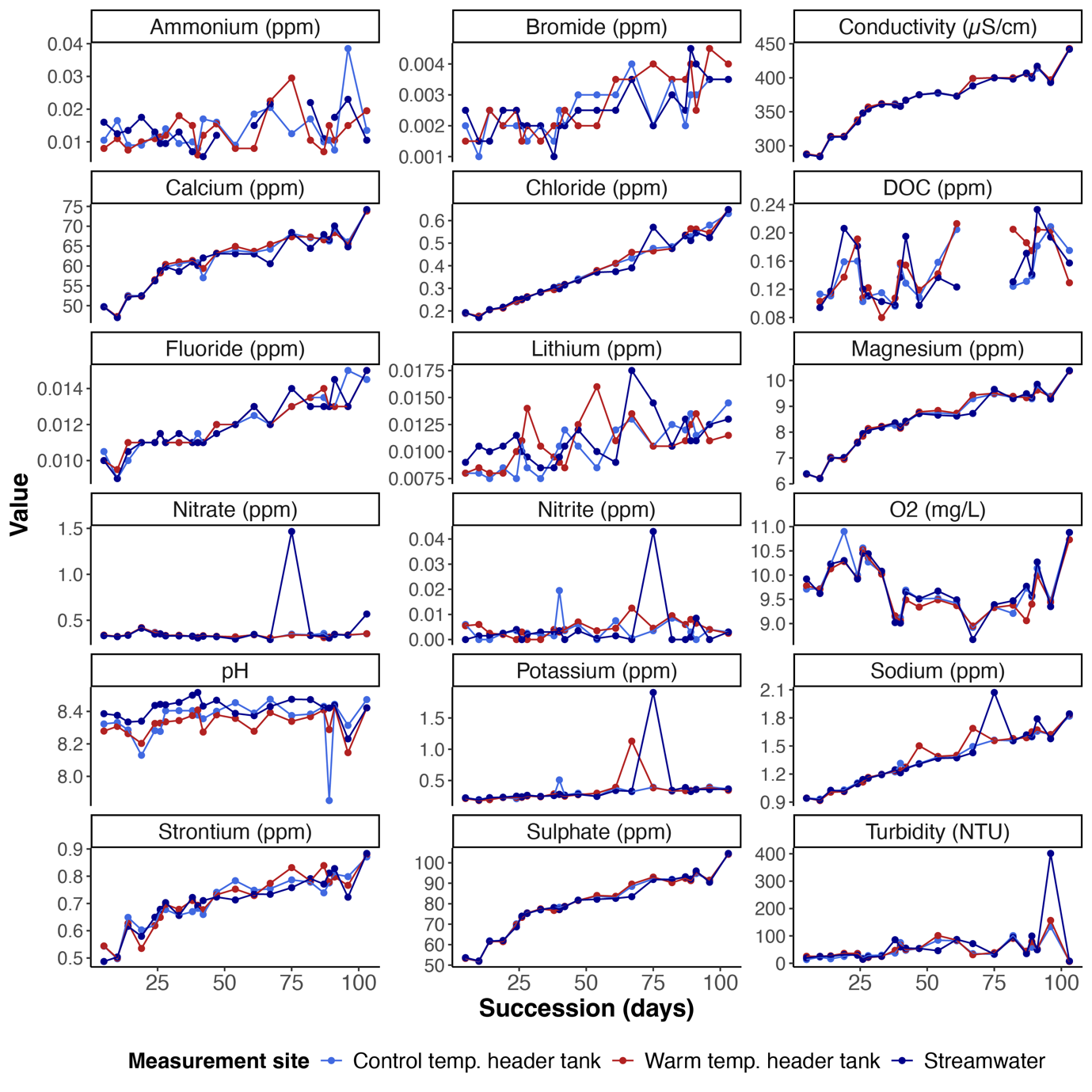


**Figure S2.** **Changes in** **measured physicochemical parameters over the course of the experiment.** These physicochemical parameters were measured in the stream water and the two header tanks.

**
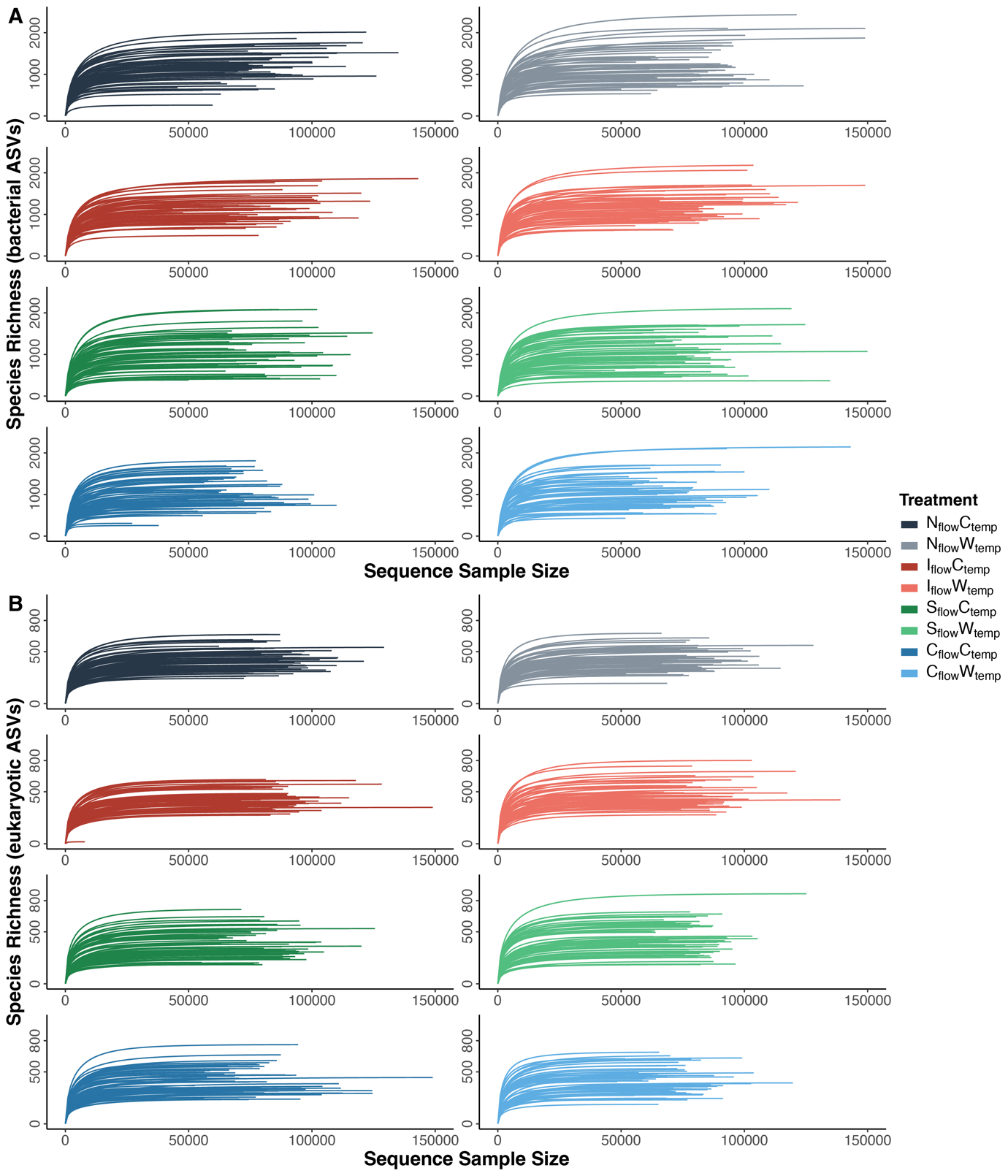
**

**Figure S3.** **Rarefaction curves showing ASV richness of glacier-fed stream biofilms. (A)** Bacterial (16S rRNA gene) and **(B)** eukaryotic (18S rRNA gene) sequencing depth per sample.

**
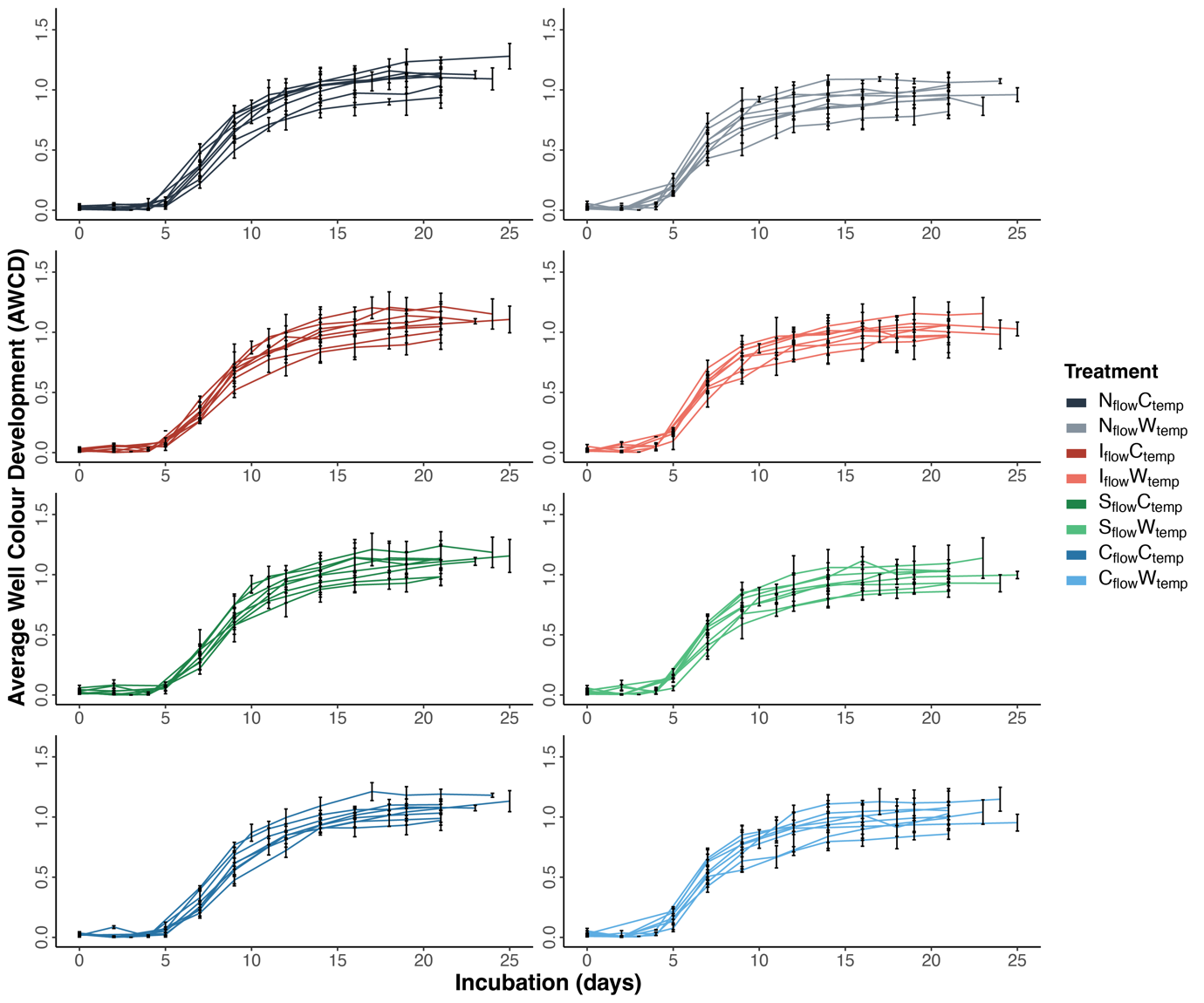
**

**Figure S4. EcoPlates average well colour development (AWCD) monitoring.** EcoPlates OD_590_ was measured three times weekly over three weeks, and AWCD was calculated each time to confirm stabilisation of metabolic activity (AWCD reaching a plateau).

**
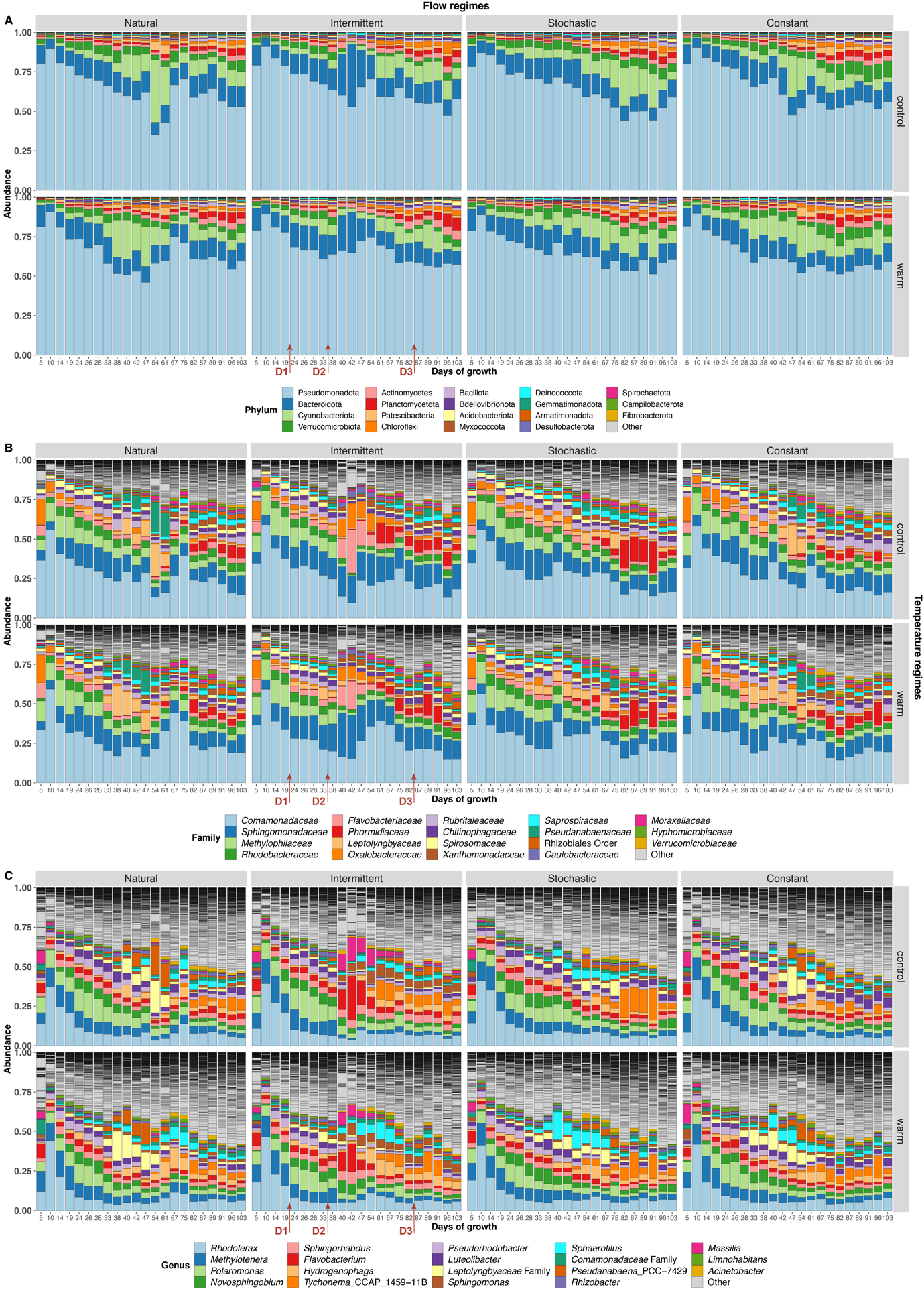
**

**Figure S5.** **Taxonomic composition of glacier-fed stream biofilm bacteria (16S rRNA gene) under climate change scenarios.** Composition at **(A)** phylum, **(B)** family, and **(C)** genus levels. D1, D2, and D3 indicate the timing of the three droughts.

**
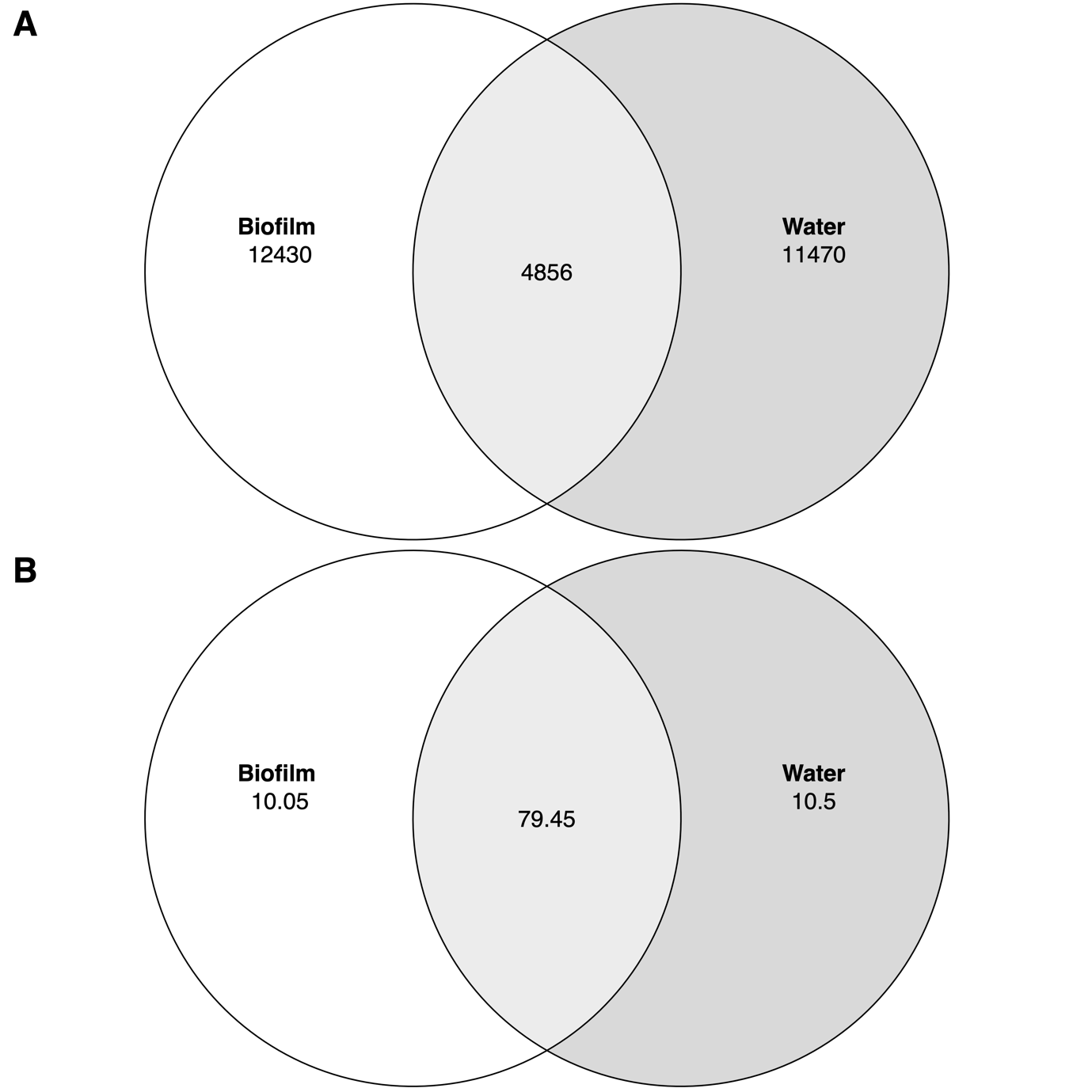
**

**Figure S6. Venn diagram of bacterial (16S rRNA gene) ASV overlap between the total biofilm and streamwater samples.** Overlap **(A)** in terms of prevalence of ASVs and **(B)** in terms of relative abundance (%).


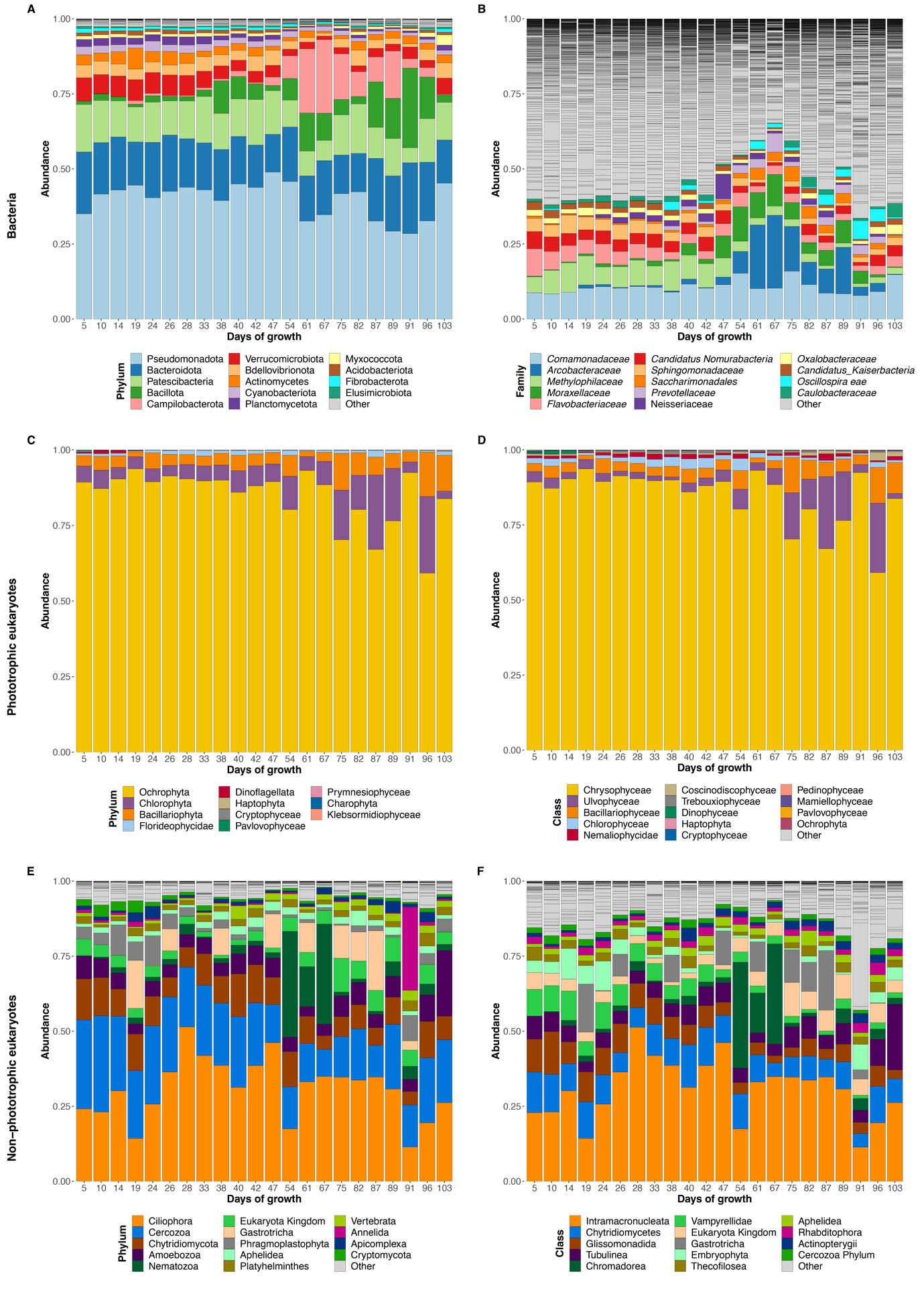


**Figure S7.** **Taxonomic composition of *Dranse de Ferret* glacier-fed stream water over the sampling campaign.** Bacterial (16S rRNA gene) composition at **(A)** phylum and **(B)** family levels. Taxonomic composition of phototrophic eukaryotes (18S rRNA gene) at **(C)** phylum and **(D)** class levels, and of non-phototrophic eukaryotes (18S rRNA gene) at **(E)** phylum, and **(F)** class levels.

**
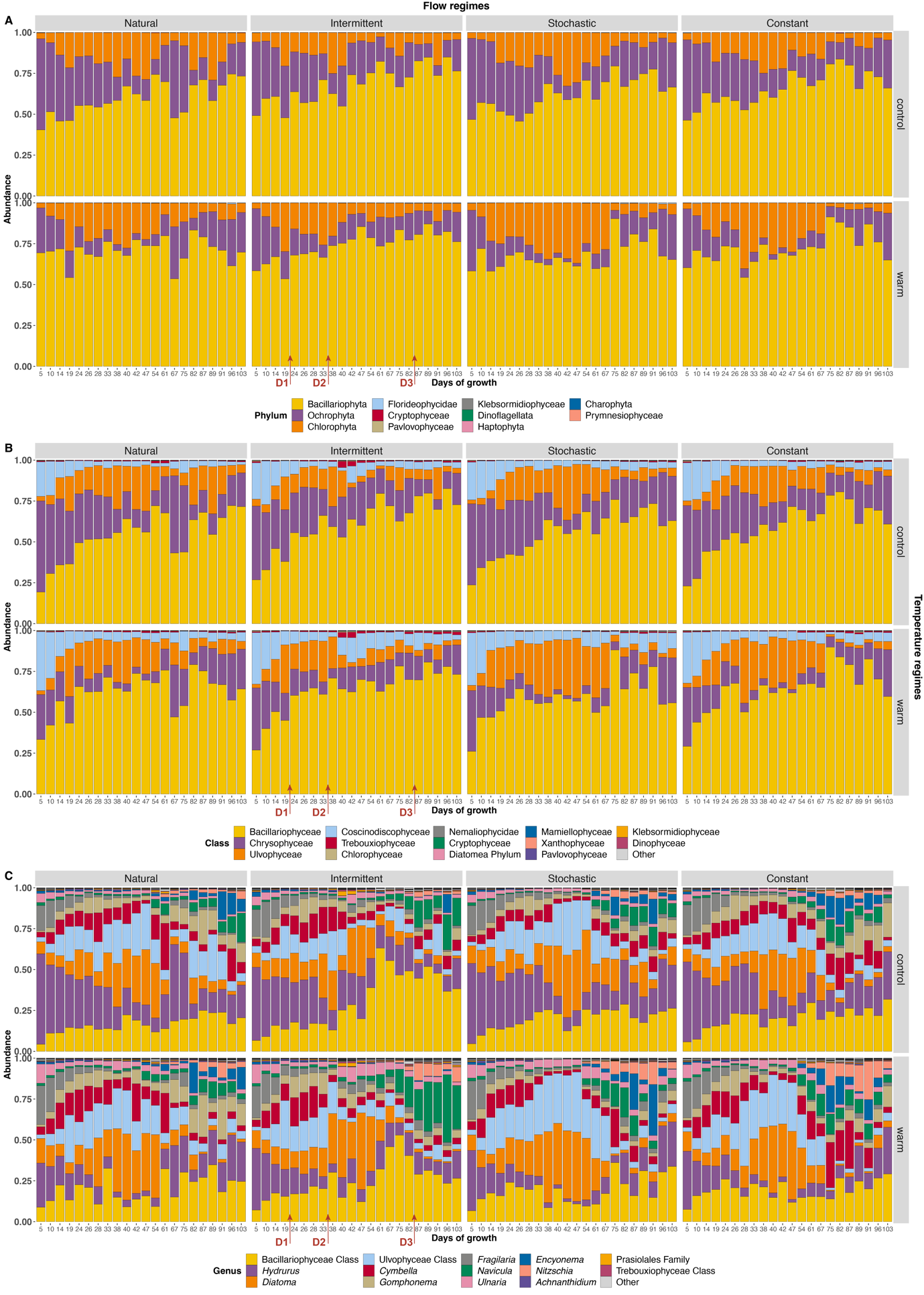
**

**Figure S8.** **Taxonomic composition of glacier-fed stream biofilm phototrophic eukaryotes (18S rRNA gene) under climate change scenarios.** Composition at **(A)** phylum, **(B)** class, and **(C)** genus levels. D1, D2, and D3 indicate the timing of the three droughts.

**
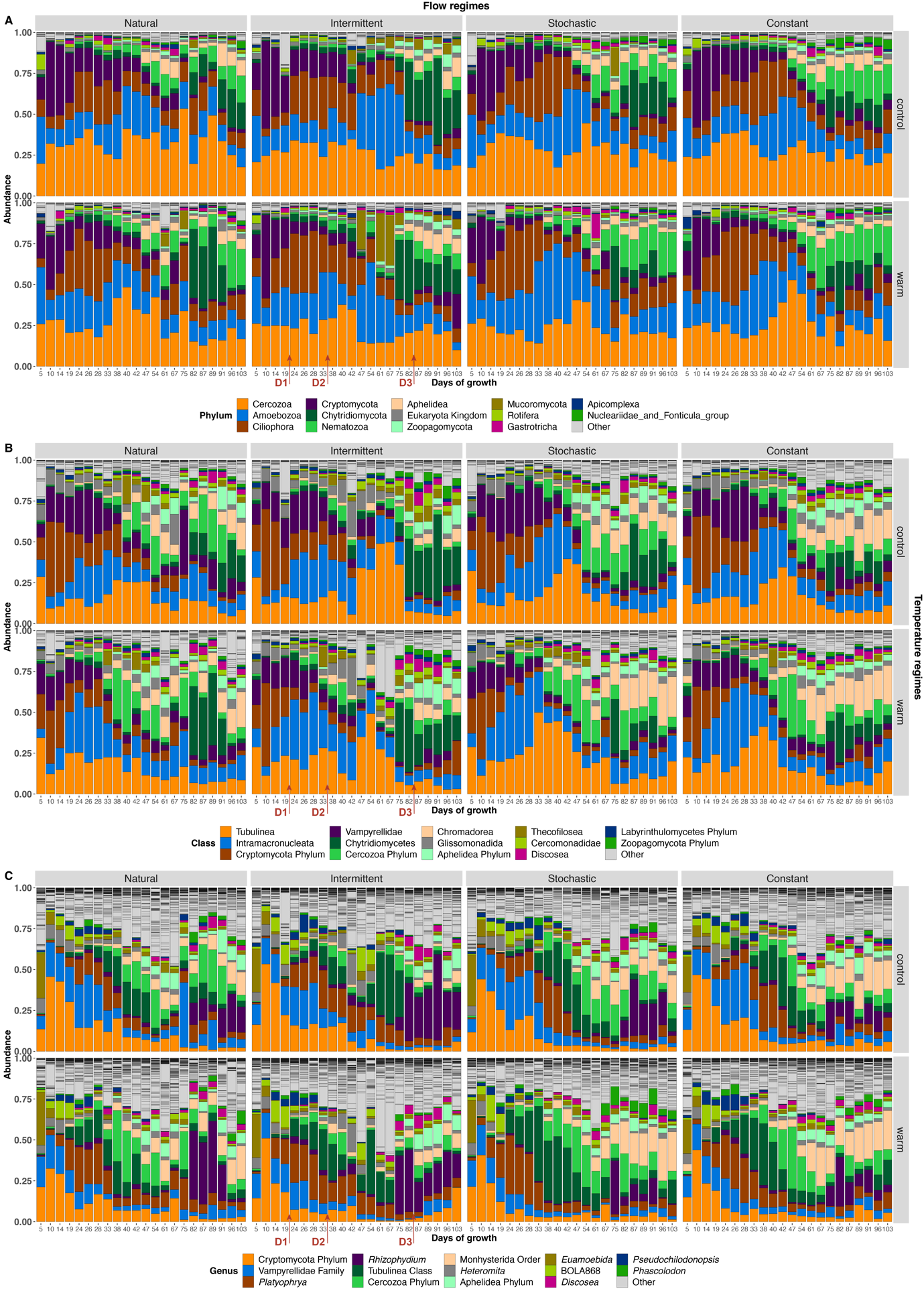
**

**Figure S9.** **Taxonomic composition of glacier-fed stream biofilm non-phototrophic eukaryotes (18S rRNA gene) under climate change scenarios.** Composition at **(A)** phylum, **(B)** class, and **(C)** genus levels. D1, D2, and D3 indicate the timing of the three droughts.


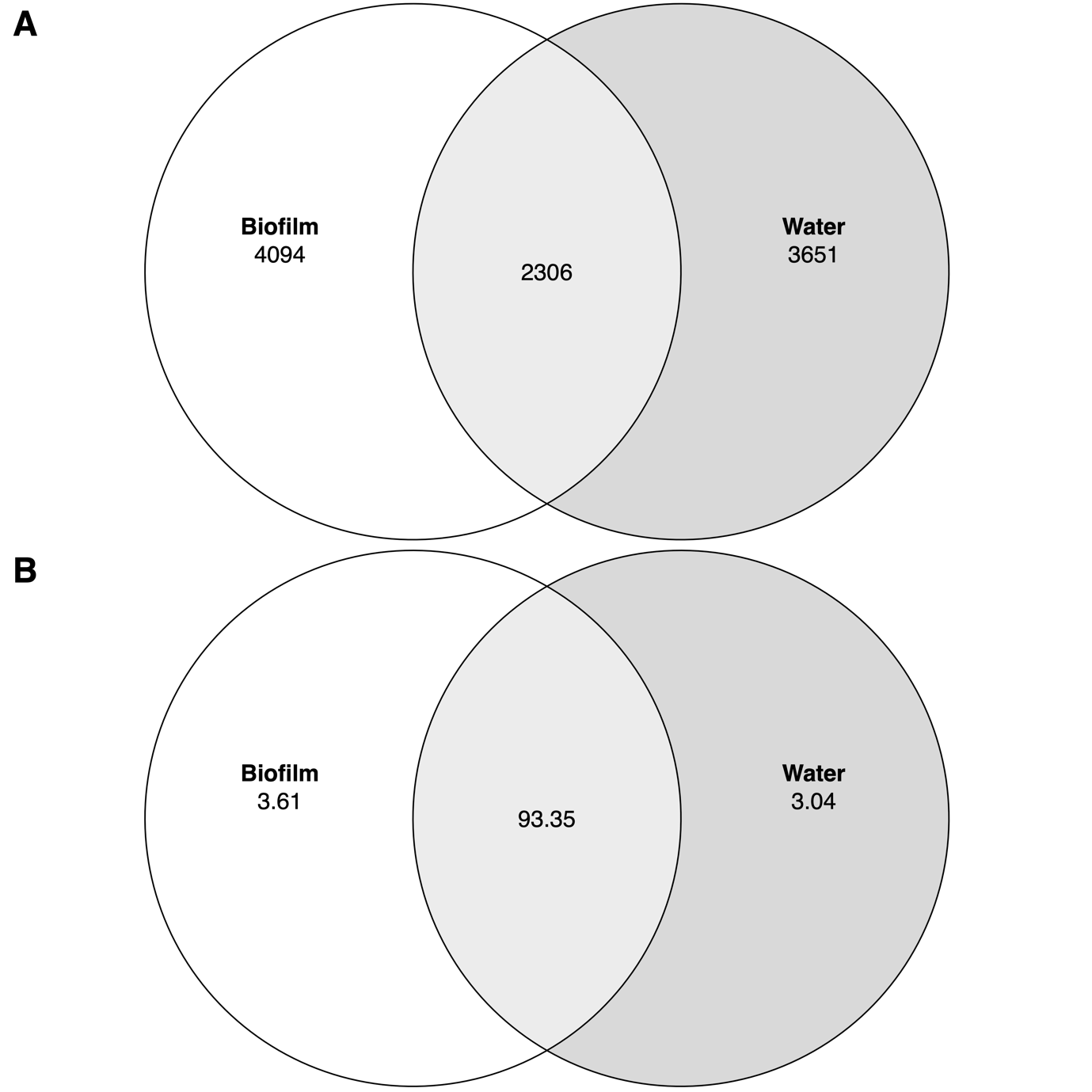


**Figure S10. Venn diagram of eukaryotic (18S rRNA gene) ASV overlap between the total biofilm streamwater samples.** Overlap **(A)** in terms of prevalence of ASVs and **(B)** in terms of relative abundance (%).


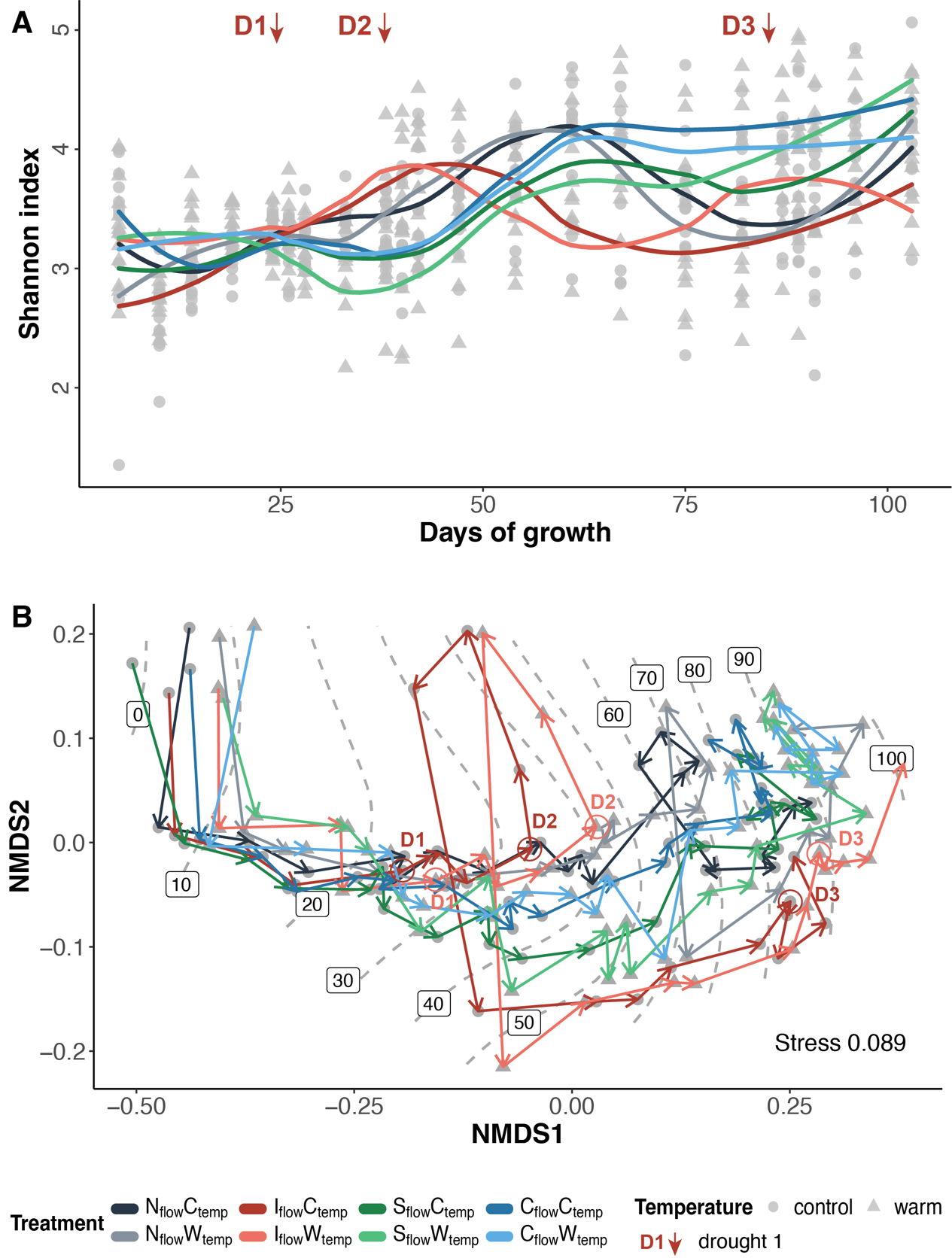


**Figure S11. Diversity and community composition of glacier-fed stream biofilm non-phototrophic eukaryote members under climate change scenarios.** **(A)** Shannon alpha diversity index of non-phototrophic eukaryotes for each treatment and time point. **(B)** Changes in non-phototrophic eukaryotic community composition illustrated by nonmetric multidimensional scaling (NMDS) ordination based on Bray-Curtis dissimilarity. Arrows indicate the ecological trajectories of biofilm samples over time. Dashed contour lines represent time-based predictions from the “ordisurf” analysis, illustrating predicted community composition over succession. Replicate samples were merged for visualisation. D1, D2, and D3 indicate the timing of the three droughts.


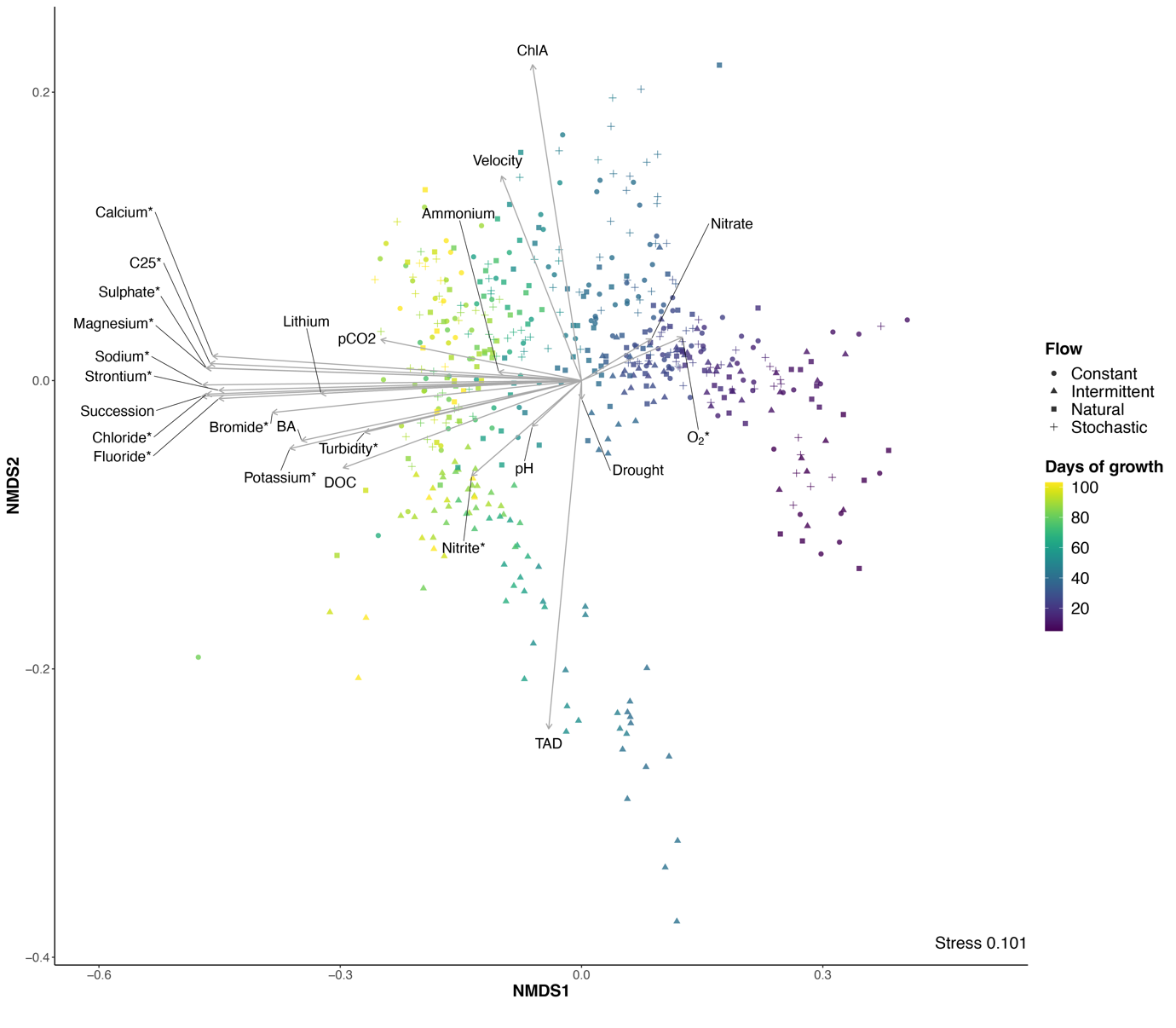


**Figure S12.** **Bacterial community composition of glacier-fed stream biofilms under climate change scenarios.** Changes in bacterial community composition shown by a nonmetric multidimensional scaling (NMDS) ordination based on Bray-Curtis dissimilarity. Arrows show environmental parameters significantly explaining changes in community composition (*p* < 0.05). BA: Bacterial abundance, ChlA: Chlorophyll-*a*, DOC: Dissolved organic carbon, TAD: Time after drought, C25: Conductivity. Stars indicate a collinearity between the environmental parameters and the “Succession” variable (VIF > 5).


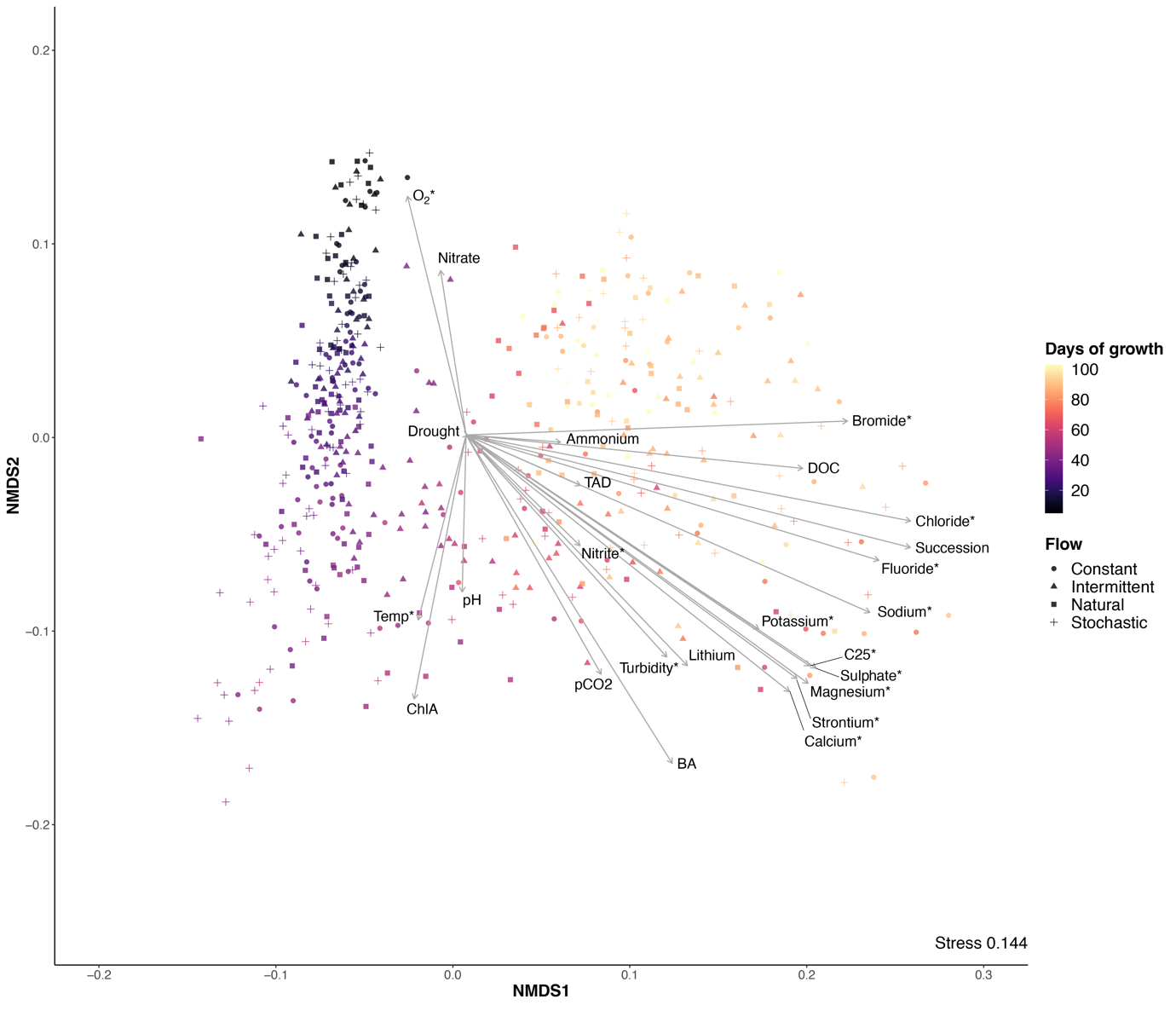


**Figure S13.** **Phototrophic eukaryotes community composition of glacier-fed stream biofilms under climate change scenarios.** Changes in phototrophic eukaryotes community composition shown by a nonmetric multidimensional scaling (NMDS) ordination based on Bray-Curtis dissimilarity. Arrows show environmental parameters significantly explaining changes in community composition (*p* < 0.05). BA: Bacterial abundance, ChlA: Chlorophyll-*a*, DOC: Dissolved organic carbon, TAD: Time after drought, C25: Conductivity. Stars indicate a collinearity between the environmental parameters and the “Succession” variable (VIF > 5).


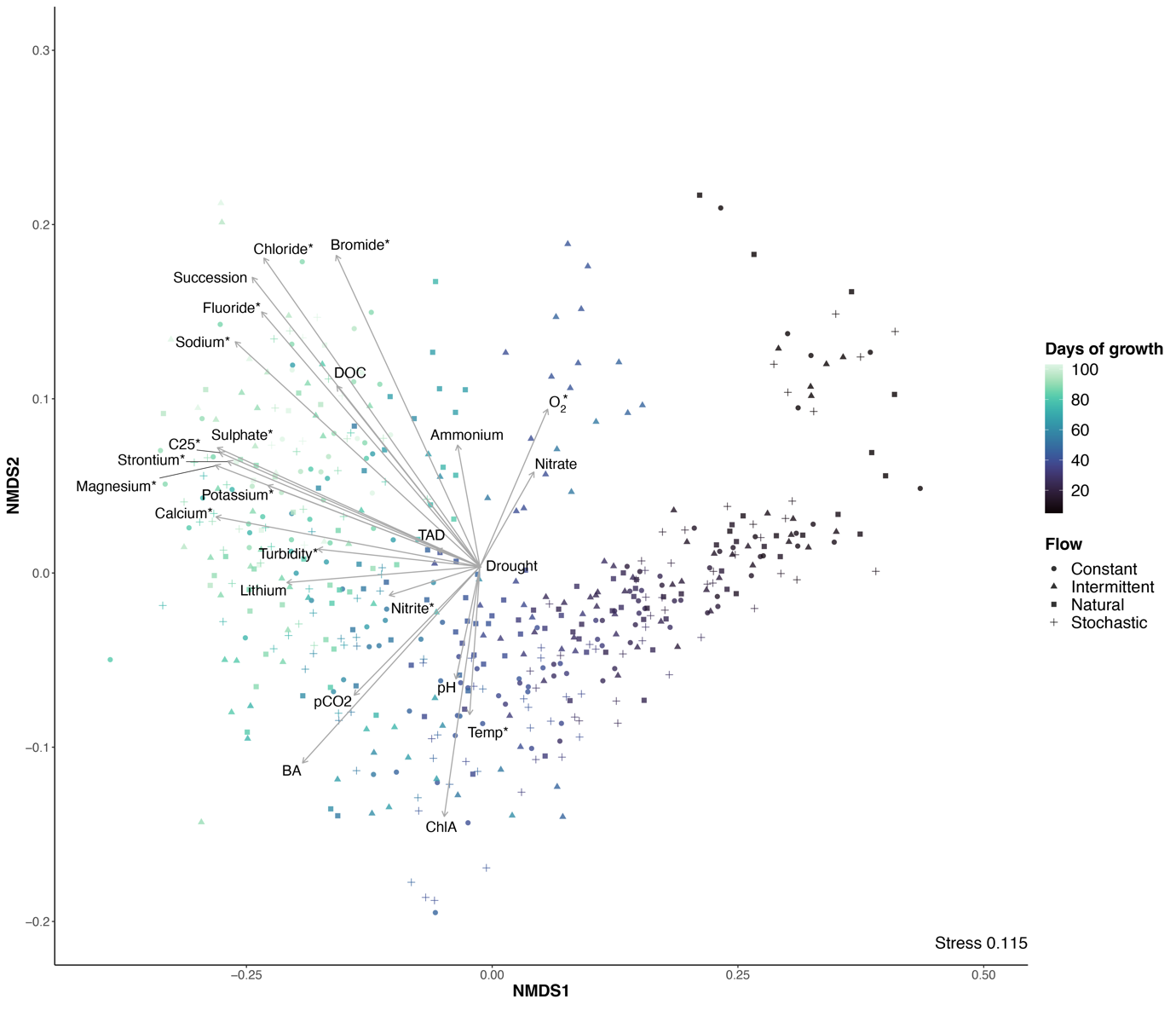


**Figure S14.** **Non-phototrophic eukaryotes community composition of glacier-fed stream biofilms under climate change scenarios.** Changes in non-phototrophic eukaryotes community composition shown by a nonmetric multidimensional scaling (NMDS) ordination based on Bray-Curtis dissimilarity. Arrows show environmental parameters significantly explaining changes in community composition (*p* < 0.05). BA: Bacterial abundance, ChlA: Chlorophyll-*a*, DOC: Dissolved organic carbon, TAD: Time after drought, C25: Conductivity. Stars indicate a collinearity between the environmental parameters and the “Succession” variable (VIF > 5).


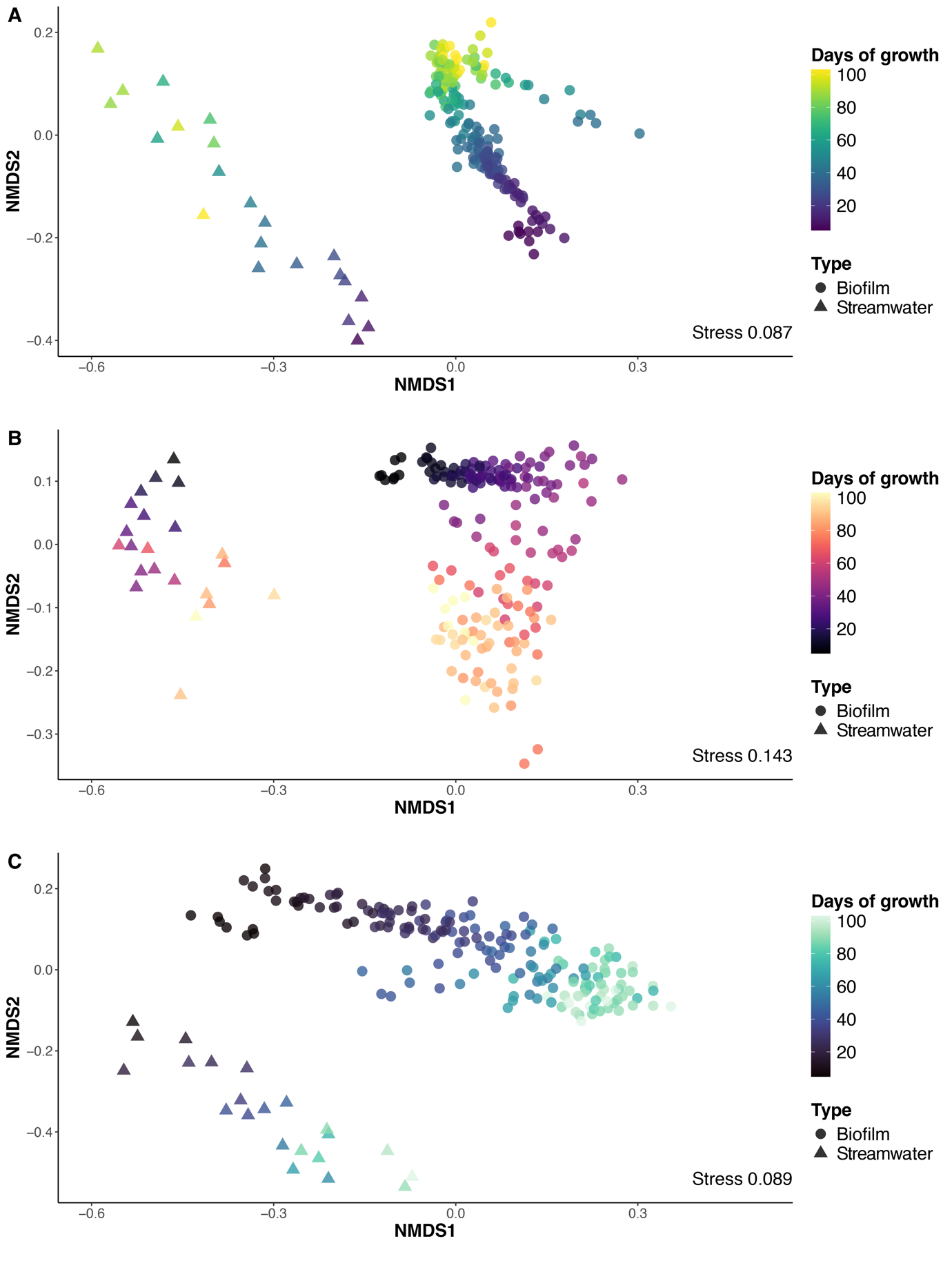


**Figure S15. Community composition of the glacier-fed stream *Dranse de Ferret* streamwater and mesocosms GFS biofilms.** Changes in community composition of **(A)** bacteria, **(B)** phototrophic eukaryotes, and **(C)** non-phototrophic eukaryotes, illustrated by nonmetric multidimensional scaling (NMDS) ordination based on Bray-Curtis dissimilarity. Replicate biofilm samples were merged for visualisation. For all microbial communities, composition significantly differed between biofilm and streamwater samples (*p* < 0.001).


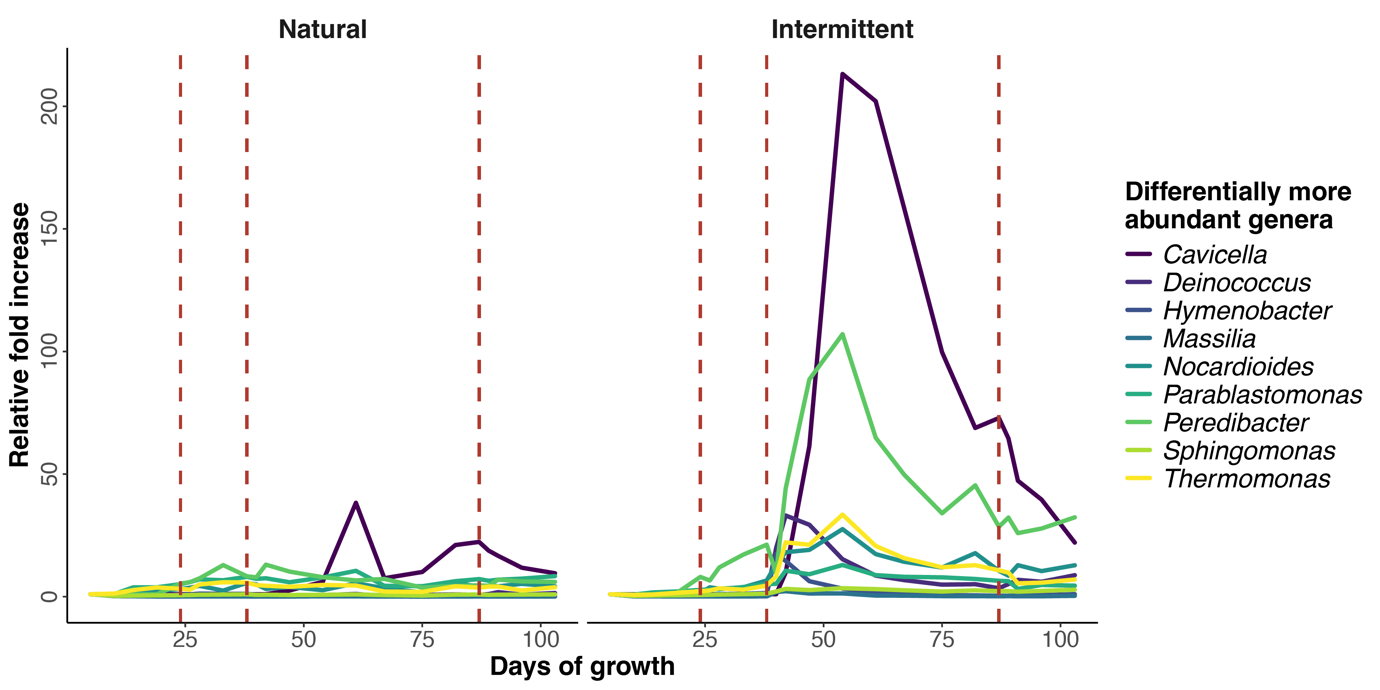


**Figure S16.** **Changes in relative abundance of the nine known differentially more abundant genera in the intermittent flow regime over time (i.e., *Deinococcus*, *Nocardioides*, *Massilia*, *Peredibacter*, *Hymenobacter*, *Cavicella*, *Thermomonas*, *Sphingomonas*, *Parablastomonas*).** The abundance values were normalized to the abundance of the first time point (day 5) to better visualize the increase in abundance over time. Note that a small amount was added to each value (+0.00001), smaller than the lowest abundance, as the *Cavicella* genus was not present in our biofilms at the first time point. Red dashed lines indicate when droughts occurred.

**
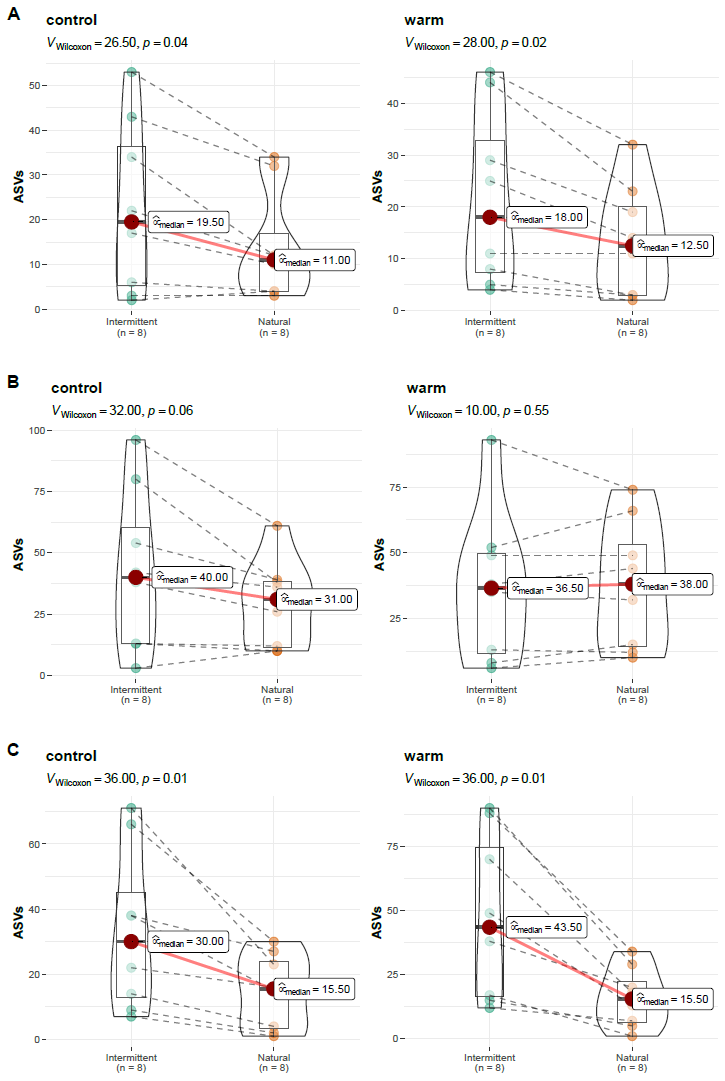
Figure S17. Dominant community assembly processes of known differentially abundant genera in the intermittent flow treatments (i.e., *Deinococcus*, *Nocardioides*, *Massilia*, *Peredibacter*, *Hymenobacter*, *Cavicella*, *Thermomonas*, and *Sphingomonas;* the genus *Parablastomonas* did not have enough ASVs across both treatments to be plotted here)*.*** Shown is the number of ASVs in bins identified to be under homogeneous selection (HoS, panel **A**), ecological drift (DR, panel **B**), and dispersal limitation (DL, panel **C**) for control and warm thermal regimes, respectively, using the iCAMP framework. Each genus is represented as a single point, and dashed lines connect the respective genus across the flow treatments (i.e., intermittent and natural flow). The median number of ASVs across all eight genera is highlighted. Compared to natural flow treatments, treatments experiencing intermittency have significantly more ASVs in phylogenetic bins associated with HoS and DR, suggesting that these community assembly processes importantly contribute to the re-structuring of communities in the face of flow intermittency. We suggest that drying exerts strong selective pressure on stream biofilm bacteria, hence explaining the importance of HoS. In contrast, DL reflects the importance of (limited) temporal persistence rather than spatial connectivity in our time-resolved experimental design. Hence, intermittency appears to importantly influence the temporal persistence of taxa that benefit from dry periods.
